# Specific ablation of CD4^+^ T-cells promotes heart regeneration in juvenile mice

**DOI:** 10.7150/thno.42943

**Published:** 2020-06-29

**Authors:** Jiatao Li, Cai Liang, Kevin Y. Yang, Xiuzhen Huang, Mao Ying Han, Xisheng Li, Vicken W. Chan, Kathleen S. Chan, Di Liu, Zhan-Peng Huang, Bin Zhou, Kathy O. Lui

**Affiliations:** 1 Department of Chemical Pathology; and Li Ka Shing Institute of Health Sciences, Prince of Wales Hospital, The Chinese University of Hong Kong, Hong Kong, China.; 2 The State Key Laboratory of Cell Biology, CAS Center for Excellence in Molecular Cell Science, Shanghai Institute of Biochemistry and Cell Biology, Chinese Academy of Sciences, University of Chinese Academy of Sciences, Shanghai, China.; 3 Department of Cardiology, Center for Translational Medicine, Institute of Precision Medicine, The First Affiliated Hospital, Sun Yat-sen University, Guangzhou, China.; 4 Lead Contact.

**Keywords:** CD4^+^ T-cells, neonatal heart regeneration, juvenile heart regeneration, cardiomyocyte proliferation, cardiac fibrosis, macrophages.

## Abstract

Unlike adult cardiomyocytes, neonatal cardiomyocytes can readily proliferate that contributes to a transient regenerative potential after myocardial injury in mice. We have recently reported that CD4^+^ regulatory T-cells promote this process; however, the role of other CD4^+^ T-cell subsets as well as CD8^+^ T-cells in postnatal heart regeneration has been less studied. **Methods:** by comparing the regenerating postnatal day (P) 3 and the non-regenerating P8 heart after injury, we revealed the heterogeneity of CD4^+^ and CD8^+^ T-cells in the myocardium through single cell analysis. We also specifically ablated CD4^+^ and CD8^+^ T-cells using the lytic anti-CD4 and -CD8 monoclonal antibodies, respectively, in juvenile mice at P8 after myocardial injury. **Results**: we observe significantly more CD4^+^FOXP3^-^ conventional T-cells in the P8 heart when compared to that of the P3 heart within a week after injury. Surprisingly, such a difference is not seen in CD8^+^ T-cells that appear to have no function as their depletion does not reactivate heart regeneration. On the other hand, specific ablation of CD4^+^ T-cells contributes to mitigated cardiac fibrosis and increased cardiomyocyte proliferation after injury in juvenile mice. Single-cell transcriptomic profiling reveals a pro-fibrotic CD4^+^ T-cell subset in the P8 but not P3 heart. Moreover, there are likely more Th1 and Th17 cells in the P8 than P3 heart. We further demonstrate that cytokines of Th1 and Th17 cells can directly reduce the proliferation and increase the apoptosis of neonatal cardiomyocytes. Moreover, ablation of CD4^+^ T-cells can directly or indirectly facilitate the polarization of macrophages away from the pro-fibrotic M2-like signature in the juvenile heart. Nevertheless, ablation of CD4^+^ T-cells alone does not offer the same protection in the adult heart after myocardial infarction, suggesting a developmental change of immune cells including CD4^+^ T-cells in the regulation of age-related mammalian heart repair. **Conclusions**: our results demonstrate that ablation of CD4^+^ but not CD8^+^ T-cells promotes heart regeneration in juvenile mice; and CD4^+^ T-cells play a distinct role in the regulation of heart regeneration and repair during development.

## Introduction

Heart disease remains as the leading cause of mortality globally in the past 15 years. After heart attack, necrotic myocardial tissue is first replaced by a non-contractile collagenous scar; and progressive remodeling of the viable cardiac muscle decreases contractile function subsequently leading to heart failure. Recent studies demonstrate that the mouse heart can transiently yet functionally regenerate after birth till postnatal day 7 (P7) 1; and functional neonatal heart regeneration is also observed in human 2. Therefore, understanding how the neonatal heart regenerates could shed light on therapeutic development targeting heart repair and regeneration. In contrast to the adult ones, neonatal cardiomyocytes readily proliferate with minimal cardiac fibrosis being observed after injury 1, 3, 4. Cellular mechanisms driving neonatal cardiomyocyte proliferation have been less studied. Recently, we have reported that CD4^+^FOXP3^+^ regulatory T-cells (Treg) can directly promote the proliferation and regeneration of blood vessels 5 and heart muscle 6 after injury, respectively. Nevertheless, the functional roles of different T-cell subsets in the regulation of neonatal heart regeneration remain unclear.

After myocardial injury, the release of neoantigens to the circulation probably activates and recruits T-cells from the lymphoid organs to the injured tissues. In adult mice, both CD4^+^ 6-9 and CD8^+^ 10, 11 T-cells have been identified in the injured myocardium that peak at around day 7 after myocardial infarction (MI) 8, 10. Nevertheless, the role of CD4^+^ T-cells in adult heart repair remains controversial. In one study, serial echocardiography showed an increased left ventricular dilation in the CD4 knockout mice compared with the wild type control for up to 56 days after MI 7, suggesting a protective role of CD4^+^ T-cells in facilitating cardiac repair. Moreover, the same group has later revealed that CD4^+^FOXP3^+^ Treg improve cardiac would healing by modulating macrophages from M1- to M2-like polarization after MI 9. Furthermore, the angiotensin-II type-2 receptor (AT2R)^+^CD4^+^ T-cells have been demonstrated to reduce infarct size and improve heart function after MI in rats 12. In another study, however, it has been demonstrated that mitigated transition of hypertrophy to heart failure was observed in MHCII deficient mice that lack CD4^+^ T-cells after chronic pressure overload 13. Similarly, another study has supported that ablation of CD4^+^ T-cells by 200 μg of the lytic GK1.5 monoclonal antibody reduced left ventricular remodeling and prevented left ventricular dilation at 8 weeks after MI 11, suggesting a detrimental role of CD4^+^ T-cells during adult heart repair. On the other hand, little has been known about the pathophysiological role of CD8^+^ T-cells in the adult heart after myocardial injury. In one study, the AT2R^-^CD8^+^ T-cells demonstrate potent cytotoxicity to adult rat cardiomyocytes; while the AT2R^+^CD8^+^ T-cells demonstrate a cardioprotective effect as adoptive transfer of these cells into the injured myocardium of rats results in reduced infarct size at 2 weeks after MI 10. Nonetheless, the heterogeneity of different T-cell subsets in the regulation of heart regeneration in young mice has not been investigated.

Recently, we have reported that the P3 neonatal mouse heart can functionally regenerate whereas the P8 juvenile heart shows cardiac fibrosis in response to the same degree of myocardial injury 6. In this study, we showed that there were significantly more CD4^+^FOXP3^-^ conventional T-cells per mg tissue of the non-regenerating P8 when compared to that of the regenerating P3 heart within the first week after injury. Surprisingly, we did not observe such a difference in the absolute number of myocardial CD8^+^ T-cells between the regenerating and non-regenerating hearts. These cells also appeared to have no detrimental effect as their depletion did not contribute to altered cardiac fibrosis nor cardiac cell mass after injury. In contrast, specific ablation of CD4^+^ T-cells by GK1.5 resulted in mitigated cardiac fibrosis and increased cardiomyocyte proliferation, suggesting that they could negatively regulate heart regeneration in the P8 mice. Mechanistically, single-cell RNA-sequencing delineated the heterogeneity of different T-cell subsets during postnatal heart regeneration; and revealed the lack of the pro-fibrotic CD4^+^ T-cell subset in the regenerating P3 heart. Moreover, there were likely less Th1 and Th17 cells in the regenerating P3 than non-regenerating P8 heart. We further demonstrated that cytokines of Th1 and Th17 cells directly regulate the survival of cardiomyocytes. Furthermore, CD4^+^ T-cells could modulate the polarization of pro-fibrotic macrophages in the heart after myocardial injury.

## Results

### CD4^+^ but not CD8^+^ T-cells are responsive to postnatal myocardial injury

**Figure 1:**
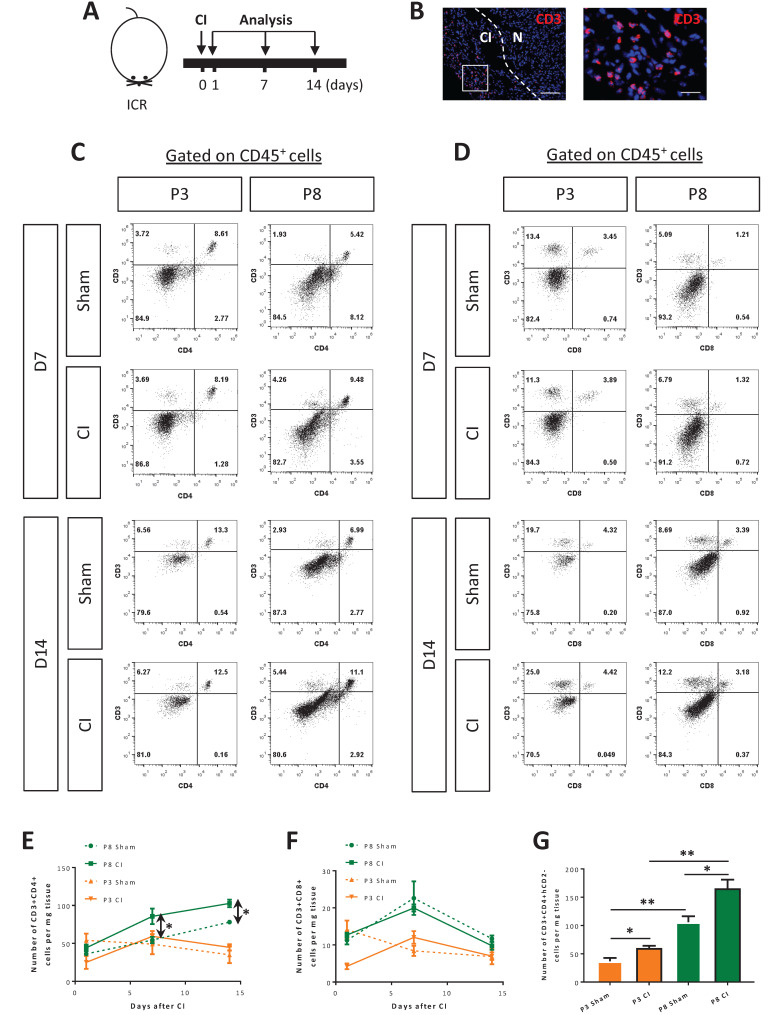
** CD4^+^ but not CD8^+^ T-cells are responsive to neonatal myocardial injury.** (A) Schematic diagram showing the experimental design for B-F. (B) Immunostaining on frozen sections for CD3^+^ (red) cells with nuclear DAPI counterstain (blue) showing infiltration of T-cells in the infarcted (CI) and normal (N) myocardium at day 1 post CI to the P3 heart, scale bar: 100 μm. Square denotes an enlarged image on the right, scale bar: 50 μm. Flow cytometric analysis showing (C) %CD3^+^CD4^+^ or (D) %CD3^+^CD8^+^ cells among total PI^-^CD45^+^ cells at days 7 and 14 after CI to the P3 or P8 heart, respectively. Quantifications of (E) C or (F) D in terms of absolute number of cells harvested per mg heart tissue at days 1, 7 and 14 after CI to the P3 or P8 heart, respectively. (G) Quantification of CD3^+^CD4^+^hCD2^-^ conventional T-cells per mg heart tissue harvested at day 7 after CI to the P3 or P8 heart of the* Foxp3^hCD2^* mice. (E-G) Data are presented as mean±S.E.M., *P<0.05, **P<0.01, n=4 per group.

To ask if activated T-cells such as conventional CD4^+^ or CD8^+^ T-cells are involved in heart regeneration during postnatal development, we performed cryoinfarction (CI) to the P3 and P8 hearts of ICR mice, respectively (Figure 1A). At as early as day 1 after CI to the P3 hearts, beyond the early phase of wound healing, we observed CD3^+^ T-cells in the infarct zone (Figure 1B). We also quantified the amount of infiltrating CD3^+^CD4^+^ and CD3^+^CD8^+^ cells by flow cytometry in the infarct zone at days 1, 7 (Figure 1C, D) and 14 (Figure 1C, D) after CI to the P3 and P8 hearts, respectively. There were significantly increased numbers of CD3^+^CD4^+^ cells at days 7 and 14 after CI to the P8 hearts compared to the respective sham controls (Figure 1E). Surprisingly, there was no significant difference in the absolute number of CD3^+^CD8^+^ cells within the first two weeks after CI to the P3 and P8 hearts compared to the respective sham controls (Figure 1F).

CD4^+^ T-cells can be sub-classified as CD4^+^FOXP3^-^ conventional and CD4^+^FOXP3^+^ regulatory cells. We have previously shown that Treg are required for driving neonatal heart regeneration 6. In this study, we focused to investigate the role of the other CD4^+^ T-cell subsets in the infarct zone of the regenerating and non-regenerating hearts, respectively. We performed CI to the P3 or P8 hearts of *Foxp3^hCD2^* mice as previously described 6 that allow us to trace CD4^+^FOXP3^-^ T-cells via the surface expression of hCD2 driven under the *Foxp3* promoter; and quantified the amount of these cells at day 7 after CI. We found that there were significantly more CD3^+^CD4^+^hCD2^-^ cells in the P8 than P3 hearts of both the injury and sham groups, indicating that the increased amount of CD4^+^ conventional T-cells could be associated with postnatal heart development (Figure 1G). Nevertheless, there were also significantly increased numbers of CD3^+^CD4^+^hCD2^-^ cells in the injury than sham groups of the P3 and P8 hearts, respectively (Figure 1G). Taken together, our results demonstrated that conventional CD4^+^ but not CD8^+^ T-cells expanded in the postnatal myocardium after injury.

### Ablation of CD4^+^ but not CD8^+^ T-cells reactivates heart regeneration after postnatal myocardial injury

**Figure 2:**
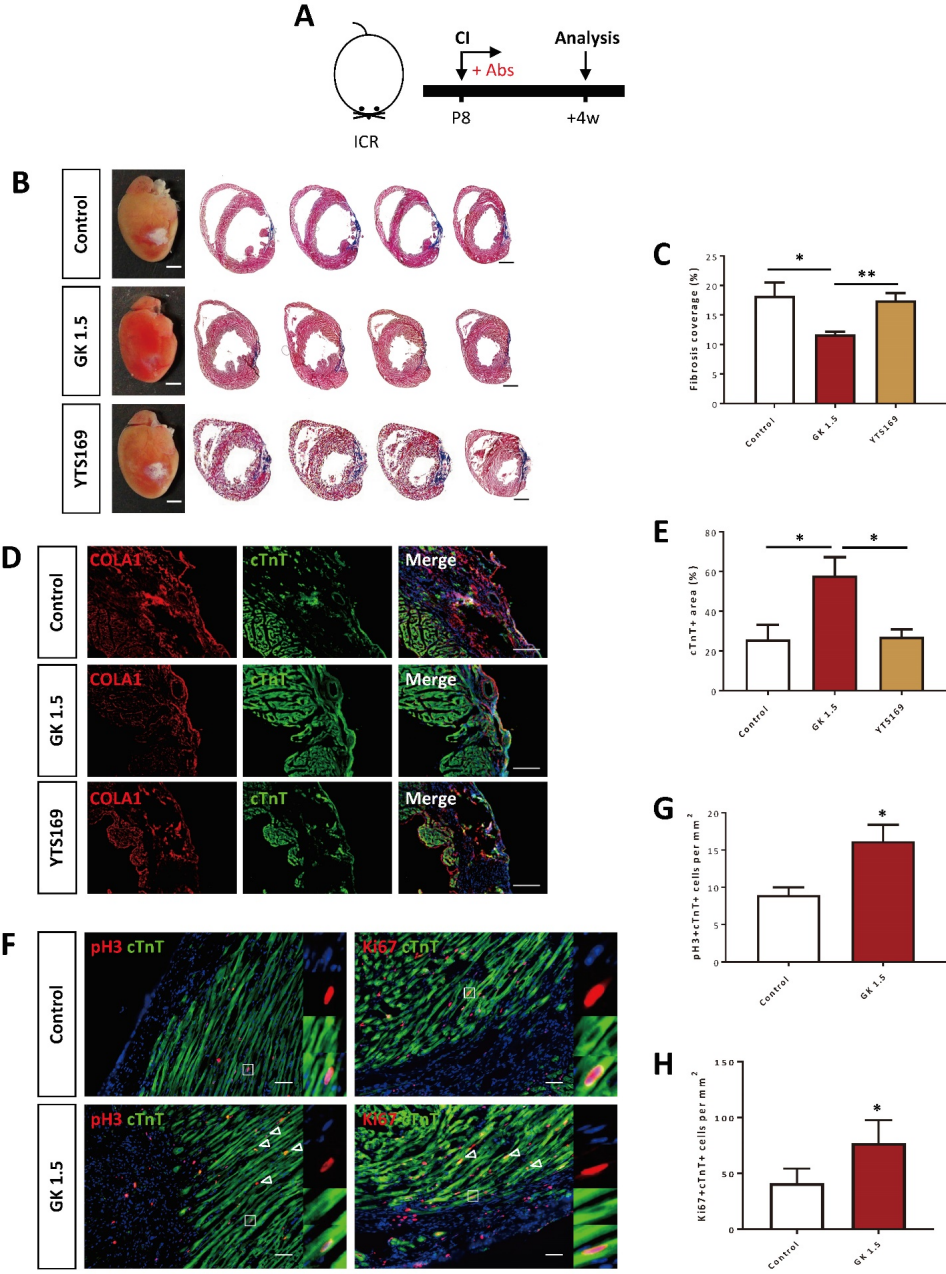
** Ablation of CD4^+^ but not CD8^+^ T-cells reactivates neonatal heart regeneration after cryoinfarction.** (A) Schematic diagram showing the experimental design. (B) Images of scar tissues, scale bars: 2000 μm; and Masson's trichrome staining showing representative serial cross sections of fibrotic tissues in blue, scale bars: 1000 μm. (C) Quantification of fibrotic tissue coverage based on (B). Immunostaining on frozen sections for (D) COLA1^+^ (red) and cTnT^+^ (green) cells within the infarct zone, scale bars: 100 μm; or (F) pH3^+^ (red) or Ki67^+^ (red) and cTnT^+^ (green) cells within the border zone, scale bars: 50 μm. (F) Arrows indicate cardiomyocytes positive for pH3 or Ki67 and squares denote magnified images on the right. Quantification of absolute number of (E) %cTnT^+^ coverage, (G) pH3^+^cTnT^+^ or (H) Ki67^+^cTnT^+^ cardiomyocytes per mm^2^ area. (C, E, G, H) Data are presented as mean±S.E.M., *P<0.05, **P<0.01, n=4-5 per group.

To study the functional role of CD4^+^ and CD8^+^ T-cells in postnatal heart regeneration, we specifically depleted them after CI to the P8 ICR heart using the lytic anti-CD4 (clone GK1.5) and -CD8 (clone YTS169) monoclonal antibodies, respectively (Figure 2A). After treatment with the respective antibodies, CD3^+^CD4^+^ or CD3^+^CD8^+^ T-cells were almost completely removed from the peripheral blood of the recipients as confirmed by flow cytometry (Figure S1). We then performed Masson's trichrome staining to identify collagen fibers formed during cardiac fibrosis at 4 weeks after CI (Figure 2B). In line with previous reports 1, 6, the control hearts failed to regenerate and showed excessive scar tissue formation (Figure 2B). Similar to the control group, treatment with YTS169 did not contribute to heart regeneration (Figure 2B). Nevertheless, treatment with GK1.5 led to significantly reduced deposition of fibrotic tissues compared to that of the control or YTS169-treated group (Figure 2C). Moreover, immunostaining of markers specific for fibroblasts and cardiomyocytes, i.e. type 1 collagen (COLA1) and cardiac troponin T (cTnT), at 4 weeks after CI demonstrated significantly reduced fibroblast deposition yet increased myocardium in the infarct zone of GK1.5 compared to that of the control or YTS169 group (Figure 2D, E). Furthermore, at day 7 after CI, costaining of cTnT and the proliferation markers phospho histone 3 (pH3) or Ki67 (Figure 2F) respectively revealed a significantly increased number of pH3^+^cTnT^+^ (Figure 2G) or Ki67^+^cTnT^+^ (Figure 2H) proliferating cardiomyocytes in the border zone of the GK1.5-treated group when compared to that of the control group. Our results suggested that ablation of CD8^+^ T-cells did not reactivate juvenile heart regeneration after CI to the P8 ICR heart. To ask if CD8^+^ T-cells regulated the survival of neonatal cardiomyocytes, we co-cultured neonatal CD8^+^ T-cells with neonatal cardiomyocytes for 3 days; and performed immunostaining for the proliferation marker pH3 and the apoptotic marker cleaved caspase 3 (cCASP3) with cTnT (Figure S2A). Our results showed that CD8^+^ T-cells did not alter the proliferation (Figure S2B) nor apoptosis (Figure S2C) of cardiomyocytes when compared to that of the PBS control, indicating that neonatal CD8^+^ T-cells were not cytotoxic to cardiomyocytes.

**Figure 3:**
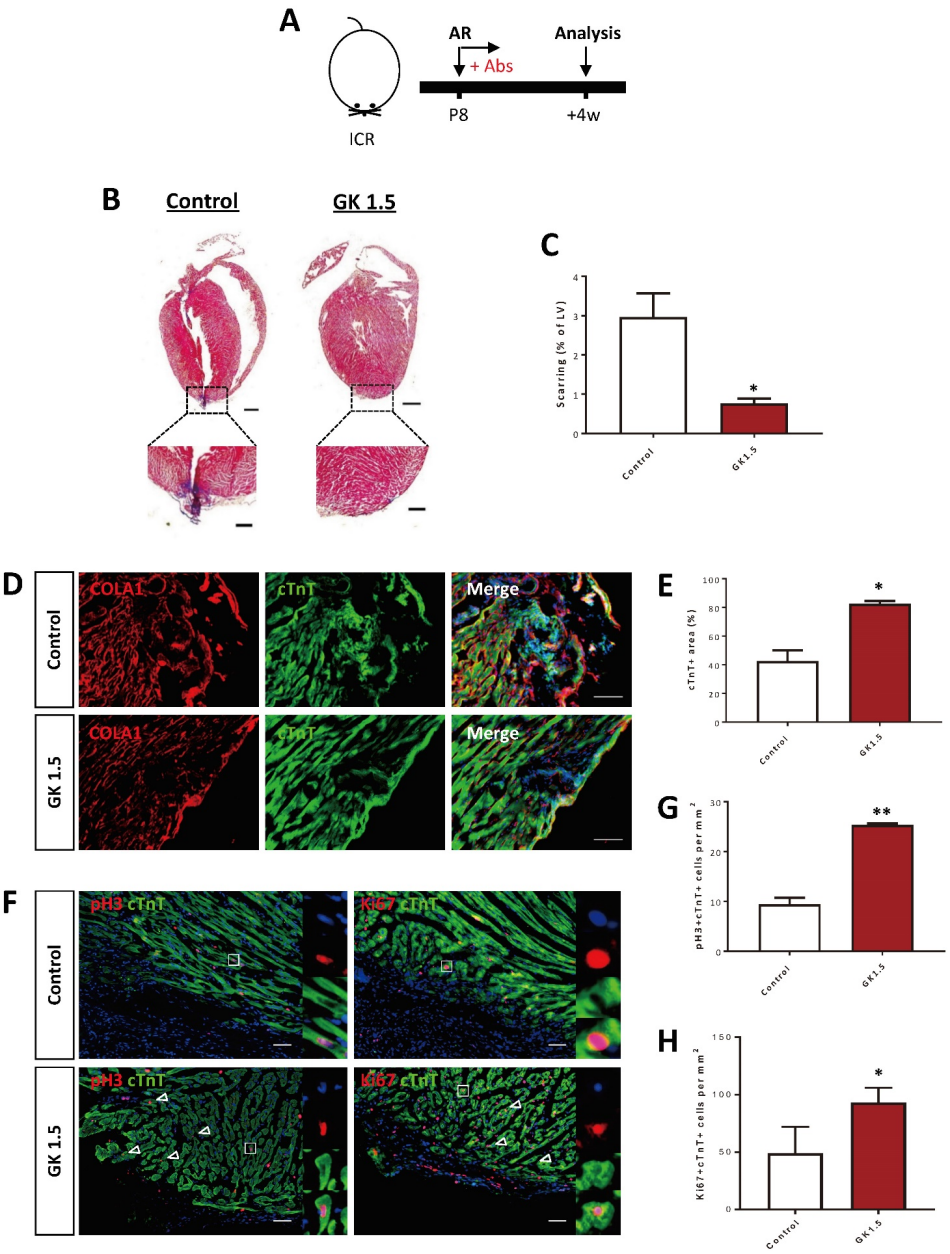
** Ablation of CD4^+^ T-cells reactivates neonatal heart regeneration after apical resection.** (A) Schematic diagram showing the experimental design. (B) Masson's trichrome staining showing representative serial cross sections of fibrotic tissues in blue, scale bars: 1000 um; and magnified images, scale bars: 200 um. (C) Quantification of scarring based on (B). Immunostaining on frozen sections for (D) COLA1^+^ (red) and cTnT^+^ (green) cells within the infarct zone, scale bars: 100 μm; or (F) pH3^+^ (red) or Ki67^+^ (red) and cTnT^+^ (green) cells within the border zone, scale bars: 50 μm. (F) Arrows indicate cardiomyocytes positive for pH3 or Ki67 and squares denote magnified images on the right. Quantification of absolute number of (E) %cTnT^+^ coverage, (G) pH3^+^cTnT^+^ or (H) Ki67^+^cTnT^+^ cardiomyocytes per mm^2^ area. (C, E, G, H) Data are presented as mean±S.E.M., *P<0.05, **P<0.01, n=4-6 per group.

We also validated the results using an additional injury model to recapitulate the therapeutic effect of CD4^+^ T-cell ablation in the reactivation of juvenile heart regeneration. We performed apical resection** (**AR) to the P8 hearts of ICR mice and examined heart regeneration at 4 weeks after AR (Figure 3A). The control but not the GK1.5-treated hearts demonstrated a significant degree of cardiac fibrosis as demonstrated by Masson's trichrome staining (Figure 3B). The resected tissues of the control hearts were replaced by scar tissues; whereas the GK1.5-treat hearts showed significantly reduced formation of fibrotic tissues when compared to that of the control hearts (Figure 3C). Immunostaining of COLA1 and cTnT at 4 weeks after AR demonstrated significantly reduced fibroblast deposition yet increased myocardium in the infarct zone of GK1.5 compared to that of the control group (Figure 3D, E). Furthermore, at day 7 after AR, costaining of cTnT and pH3 or Ki67 (Figure 3F) respectively revealed a significantly increased number of pH3^+^cTnT^+^ (Figure 3G) or Ki67^+^cTnT^+^ (Figure 3H) proliferating cardiomyocytes in the border zone of the GK1.5-treated group when compared to that of the control group. Altogether, our results showed that ablation of CD4^+^ but not CD8^+^ T-cells was sufficient to reactivate juvenile heart regeneration by reducing cardiac fibrosis and promoting cardiomyocyte proliferation after myocardial injury.

### Single-cell transcriptomics reveal the lack of a pro-fibrotic CD4^+^ T-cell subset in the regenerating postnatal heart after injury

**Figure 4:**
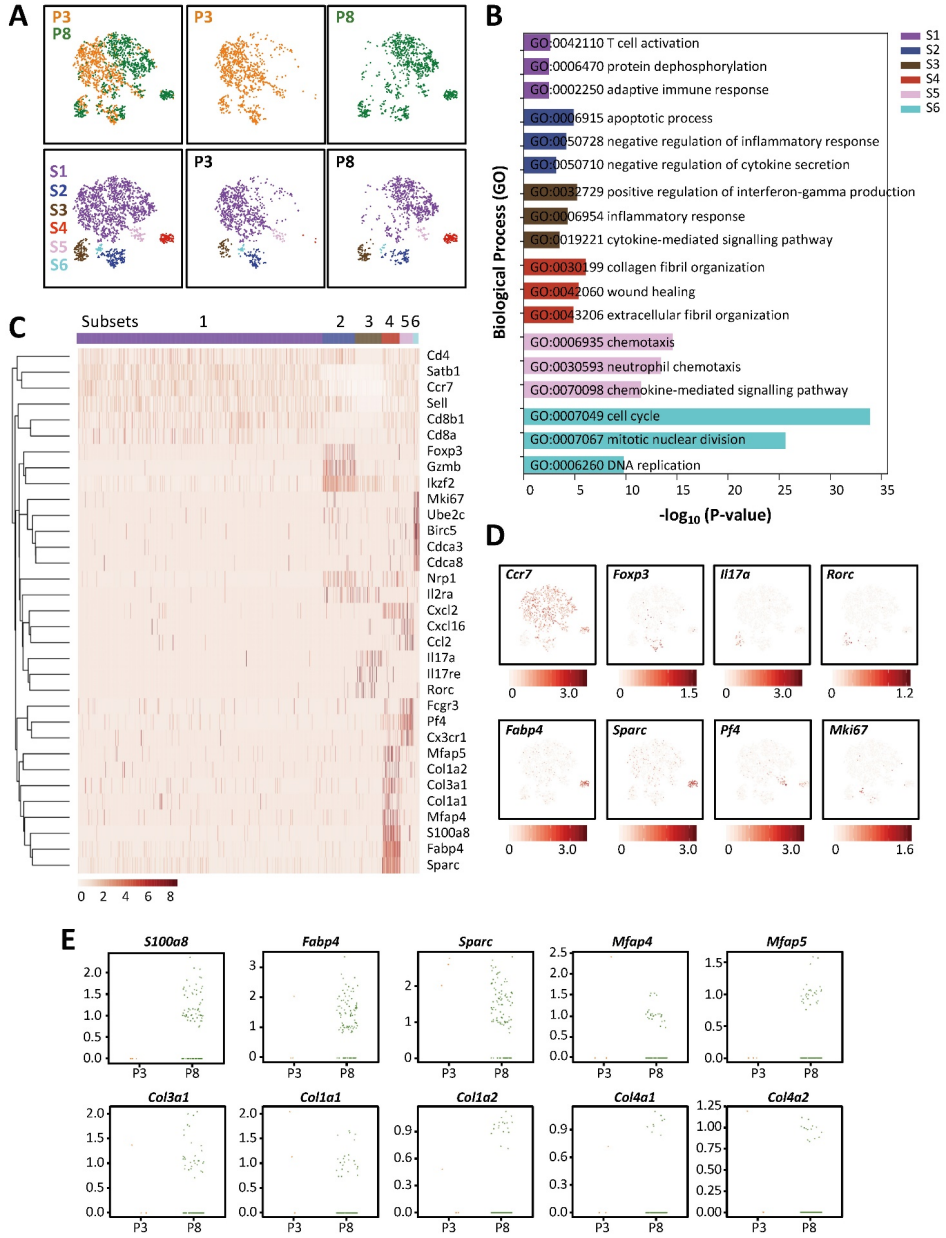
** Single-cell transcriptomics reveal the lack of a pro-fibrotic CD4^+^ T-cell subset in the regenerating neonatal heart after injury.** (A) Biaxial scatter plots by *t*-SNE analysis showing single-cell transcriptomic clustering of CD3^+^ T-cells purified at day 7 post CI to P3 and P8 hearts, respectively, by flow cytometry. Cells are subgrouped into specific subsets (S1-6). (B) Analysis of subset-specific genes showing the top 3-5 most significantly upregulated pathways determined by GO functional annotations in terms of biological processes (Table S1). (C) Upregulated genes in (B) are selectively displayed by a heatmap. (D) *t*-SNE plots showing specific genes expressed by cells that represent each of the six subsets: *Ccr7*^+^ memory cells, *Foxp3*^+^ Treg, *Il17a*^+^*Rorc*^+^ Th17 cells, *Fabp4*^+^*Sparc*^+^ pro-fibrotic cells, *Pf4*^+^ chemotactic cells and *Mki67*^+^ proliferating cells. (E) Scattered plots showing selected most significantly upregulated genes in S4 cells of the P3 and P8 hearts after CI that regulate fibroblast activation and fibrosis.

In order to understand the heterogeneity of different T-cell subsets of the regenerating and non-regenerating hearts in response to myocardial injury, we performed droplet-based genome-wide single-cell transcriptomic profiling (scRNA-seq) as recently described by us 6, 14, 15. At day 7 after injury, we purified CD45^+^CD3^+^ T-cells from the hearts of ICR mice that underwent CI at P3 and P8 by flow cytometry; and captured the transcriptomic landscape of about ~1,123 and ~2,167 cells by scRNA-seq, respectively. We performed unsupervised analysis of ~1,000 randomly selected cells from each sample. *t*-distributed stochastic neighbor embedding (*t*-SNE) plots showed that most cells expressed the *Cd3* transcripts with signature of CD4^+^ (*Cd4*, *Zbtb7b*) or CD8^+^ (*Cd8a*, *Cd8b1*) T-cells (Figure S3); and six distinct populations were identified (Figure 4A). Since our *in vivo* loss-of-function and *in vitro* cell culture experiments suggested that CD8^+^ T-cells might not play any role in the regulation of postnatal heart regeneration, we first examined if they exhibited the cytotoxic function after injury with reference to expression of cytotoxic granules. Interestingly, we observed that only very few cells expressed the cytotoxic genes such as granzymes (*Gzma*, *Gzmb*, *Gzmk*), perforin (*Prf1*) and Fas-ligand (*Fasl*); and S2 was the main subset showing *Gzmb* expression but the cells were negative for *CD8a* or* CD8b1* (Figure S4). In contrast to their response after viral infection, our cell culture (Figure S2) and scRNA-seq (Figure S4) analyses suggested that neonatal CD8^+^ T-cells were not cytotoxic to cardiomyocytes after myocardial injury.

To further annotate the identity of the six subsets, we performed pathway analysis using gene-sets that were overexpressed in each of the six subsets (Table S1). Functional annotation by Gene ontology (GO) suggested that the most significantly upregulated genes were associated with T-cell activation and adaptive immune response in S1 cells; negative regulation of inflammatory response and cytokine secretion in S2 cells; positive regulation of IFN-γ production, inflammatory and cytokine-mediated response in S3 cells; collagen and extracellular fibril organization and wound healing in S4 cells; chemotaxis and chemokine-mediated response in S5 cells; as well as cell division and DNA replication in S6 cells (Figure 4B, Table S2). Specific genes upregulated in each subset were further highlighted by heat map (Figure 4C). Based on these findings, we interpreted that most of the *Ccr7^+^Sell^+^Cd27^+^* S1 cells were probably memory T-cells (Figure S5); most of the *Foxp3^+^Ikzf2^+^Nrp1^+^Il2ra^+^* S2 cells were possibly Treg (Figure S6A); most of the *Il17a^+^Il17re^+^Rorc^+^Ccr2^+^* S3 cells were likely Th17 cells (Figure S6B); most of the *Fabp4^+^Sparc^+^* and collagen-expressing S4 cells were theoretically pro-fibrotic cells; most of the *Pf4^+^* and chemokine-expressing S5 cells were potentially chemotactic cells; and most of the *Mki67^+^* and mitotic gene-expressing S6 cells were certainly proliferating cells (Figure 4D). In addition to Th17 cells that formed a distinct subset, we also observed few Th1 (Figure S6C) and very few Th2 (Figure S6D) cells respectively located in S1, S3 and S4.

Next, we compared the heterogeneous T-cell populations of the regenerating and non-regenerating hearts. We found that there were more S3 and S4 cells but less S6 cells in the P8 when compared to that of the P3 hearts (Table 1). Additionally, S4 was a prominent subset that was enormously increased in the P8 (10.2%) compared to the P3 (0.3%) hearts (Table 1). In particular, there were a lot more P8 than P3 cells expressing genes that encoded for extracellular matrix proteins or secreted proteins associated with fibroblast activation and fibrosis such as *S100a8* 16, *Fabp4* 17, *Sparc* 18, *Mfap4* 19 and *Mfap5* 20; or collagen proteins such as *Col3a1*, *Col1a1*, *Col1a2*, *Col4a1* and *Col4a2* (Figure 4E). In the cardiovascular system, FABP4 has been demonstrated to cause pressure overload-induced cardiac hypertrophy 21; and can be therapeutically targeted against severe atherosclerosis 22. Furthermore, IFN-γ^+^ Th1 cells have been found to orchestrate cardiac fibrosis in patients with heart failure 23. Altogether, our results demonstrated that there were less Th17 cells in the regenerating than non-regenerating hearts; and T-cells could be a source of pro-fibrotic factors potentially regulating cardiac fibrosis in response to myocardial injury.

### Cytokines of CD4^+^ Th1 and Th17 cells can directly regulate the survival of postnatal cardiomyocytes

**Figure 5:**
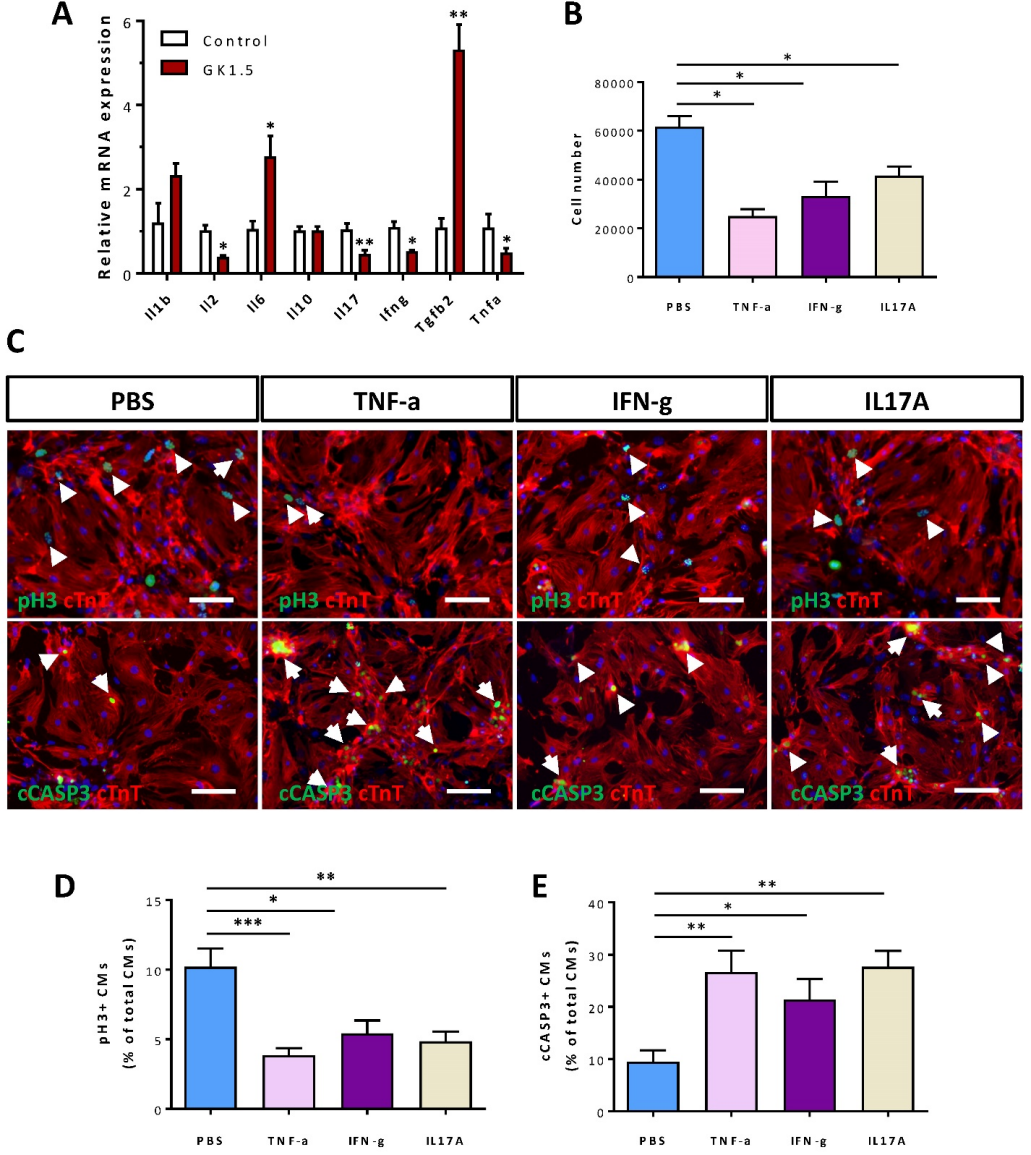
** Cytokines of Th1 and Th17 cells directly regulate the survival of neonatal cardiomyocytes.** (A) Gene expression levels of pro- and anti-inflammatory cytokines of the infarct tissues at day 7 post CI to the P8 heart as determined by qRT-PCR, data are presented as mean±S.E.M., *P<0.05, **P<0.01, n=5 per group. (B) Quantification of the absolute number of total cardiomyocytes derived from the P1 heart after cultured with PBS (solvent control), 50 ng/ml TNF-α, 50 ng/ml IFN-γ or 100 ng/ml IL-17A for 3 days. (C) Immunocytochemistry for cTnT^+^ (red) and pH3^+^ (green) or cCASP3^+^ (green) cells after cultured with PBS (solvent control), 50 ng/ml TNF-α, 50 ng/ml IFN-γ or 100 ng/ml IL-17A for 1 day, scale bars: 50 μm. White arrows indicate proliferating or apoptotic cTnT^+^ cells. (D, E) Quantification of (D) %pH3^+^cTnT^+^ or (E) cCASP3^+^cTnT^+^ cardiomyocytes among total cTnT^+^ cardiomyocytes based on (C). (B, D, E) Data are presented as mean±S.E.M., n = 3 independent experiments with a total of ~20,000 cardiomyocytes counted, *P<0.05, **P<0.01.

Our previous work showed that CD4^+^ Treg promote neonatal cardiomyocyte proliferation through paracrine factors including cytokines 6, we hypothesized that CD4^+^ T-cells such as Th1 and Th17 cells could also negatively regulate the survival of postnatal cardiomyocytes after injury in a paracrine manner. To ask if the non-regenerating juvenile heart became more inflammatory after injury, we examined the gene expression levels of both pro- and anti-inflammatory cytokines at day 7 after CI to the P8 hearts by qRT-PCR. Compared to the control heart, there were significantly reduced levels of pro-inflammatory *Il2*, *Il17*, *Ifng* and *Tnfa* while significantly increased level of anti-inflammatory *Tgfb2* in the infarct/border zones of the GK1.5-treated heart (Figure 5A). Essentially, IL-2, TNF-a, IFN-γ were produced by Th1 and IL-17 by Th17 cells 24. To ask whether these pro-inflammatory cytokines can negatively regulate the survival of postnatal cardiomyocytes, we cultured P1 cardiomyocytes with cytokines of Th1 (TNF-α or IFN-γ) and Th17 (IL-17A) cells for 1-3 days. Our results showed that TNF-α, IFN-γ, or IL-17A significantly reduced the total number of cardiomyocytes after cultured for 3 days when compared to that of the solvent control (PBS, Figure 5B). We also performed immunostaining for the proliferation marker pH3 and the apoptotic marker cCASP3 or TUNEL with cTnT (Figure 5C, Figure S7). We revealed that TNF-α, IFN-γ, or IL-17A significantly reduced %pH3^+^cTnT^+^ (Figure 5D) and increased cCASP3^+^cTnT^+^ (Figure 5E) or TUNEL^+^cTnT^+^ (Figure S7) cells among total cTnT^+^ cardiomycytes after cultured for 1 day. Collectively, our results may suggest that CD4^+^ Th1 and Th17 cells directly inhibited the proliferation and promoted the apoptosis of neonatal cardiomyocytes in a paracrine manner.

### Ablation of CD4^+^ T-cells contributes to skewed the polarization of myocardial macrophages away from the pro-fibrotic M2-like signature after injury

**Figure 6:**
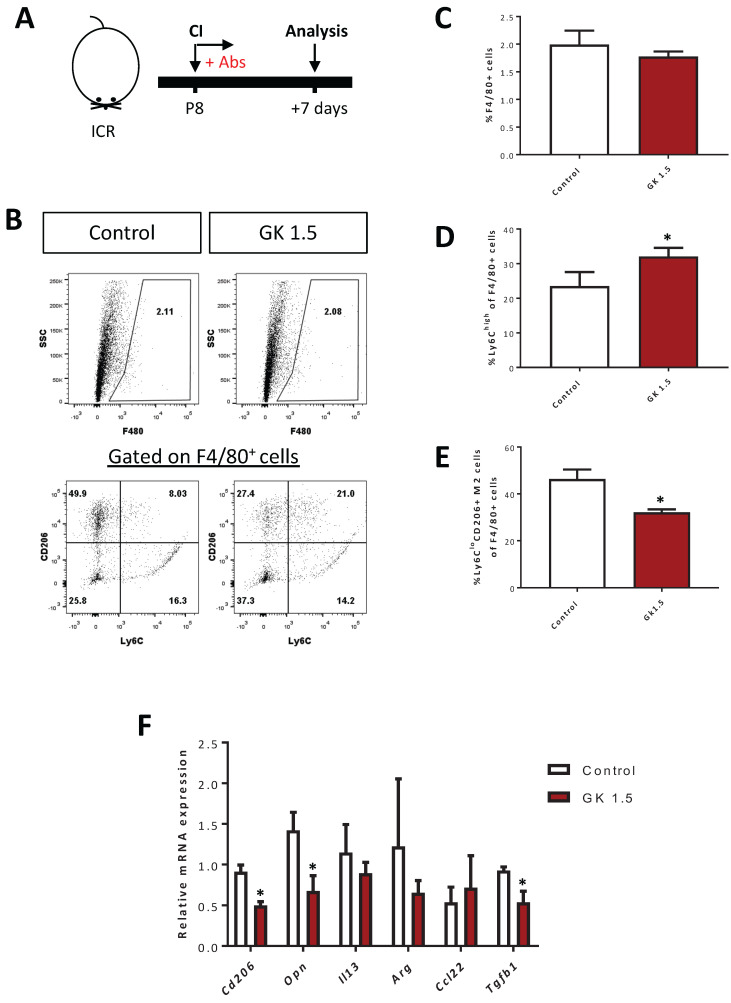
** Ablation of CD4^+^ T-cells contributes to skewed polarization of the myocardial macrophages away from the pro-fibrotic M2-like signature after injury.** (A) Schematic diagram showing the experimental design. (B) Flow cytometric analysis showing M1- or M2-like macrophages among total PI^-^F4/80^+^ macrophages at day 7 after CI to the P8 heart. Quantifications of (B) in terms of (C) %F4/80^+^ cells and (D) %Ly6C^hi^ M1- or (E) %Ly6C^lo^CD206^+^ M2-like cells among total F4/80^+^ cells harvested from the non-depleted (control) or CD4^+^ T-cell depleted (GK1.5) hearts, respectively. (F) qRT-PCR showing M2-like macrophage-associated genes of total F4/80^+^ cells purified from the control and GK1.5-treated hearts, respectively, by flow cytometry. (C-F) Data are presented as mean±S.E.M., *P<0.05, n=4-5 per group.

We have previously shown that CD4^+^ Treg promote neonatal heart regeneration by skewing the polarization of myocardial macrophages away from the pro-fibrotic M2-like signature after CI 6. Therefore, we asked if CD4^+^ T-cells also regulate macrophage polarization by examining the proportion of F4/80^+^Ly6C^hi^ M1- and F4/80^+^Ly6C^lo^CD206^+^ M2-like macrophages in the infarct/border zones after ablation of CD4^+^ T-cells at day 7 after CI to the P8 heart (Figure 6A). Our flow cytometric analyses showed that there was no significant difference in %F4/80^+^ macrophages of the GK1.5-treated and control groups (Figure 6B, C). However, there was a significantly greater percentage of the F4/80^+^Ly6C^hi^ M1-like (Figure 6B, D) but reduced percentage of the F4/80^+^Ly6C^lo^CD206^+^ M2-like (Figure 6B, E) macrophages among all F4/80^+^ macrophages of the GK1.5-treated when compared to that of the control group. Furthermore, we purified F4/80^+^ macrophages from the infarct/border zones of injured hearts of the GK1.5-treated and control groups by flow cytometry; and examined the expression levels of M2-like macrophage-associated genes including *Cd206*, *Opn* (osteopontin), *Il13* (interleukin-13), *Arg* (arginase), *Ccl22* (CC motif chemokine 22) and *Tgf-b* (transforming growth factor beta) by real-time RT-RT-qPCR. Our results demonstrated that there were significantly reduced expression levels of *Cd206*, *Opn* and *Tgfb1* in macrophages of the GK1.5-treated than control group (Figure 6F). Altogether, our results suggested that ablation of CD4^+^ T-cells did not change the quantity of macrophages but skewed their polarization away from the pro-fibrotic M2-like macrophages in the infarct zone of the P8 heart after injury.

### Ablation of CD4^+^ T-cells did not reactivate adult heart repair after injury

**Figure 7:**
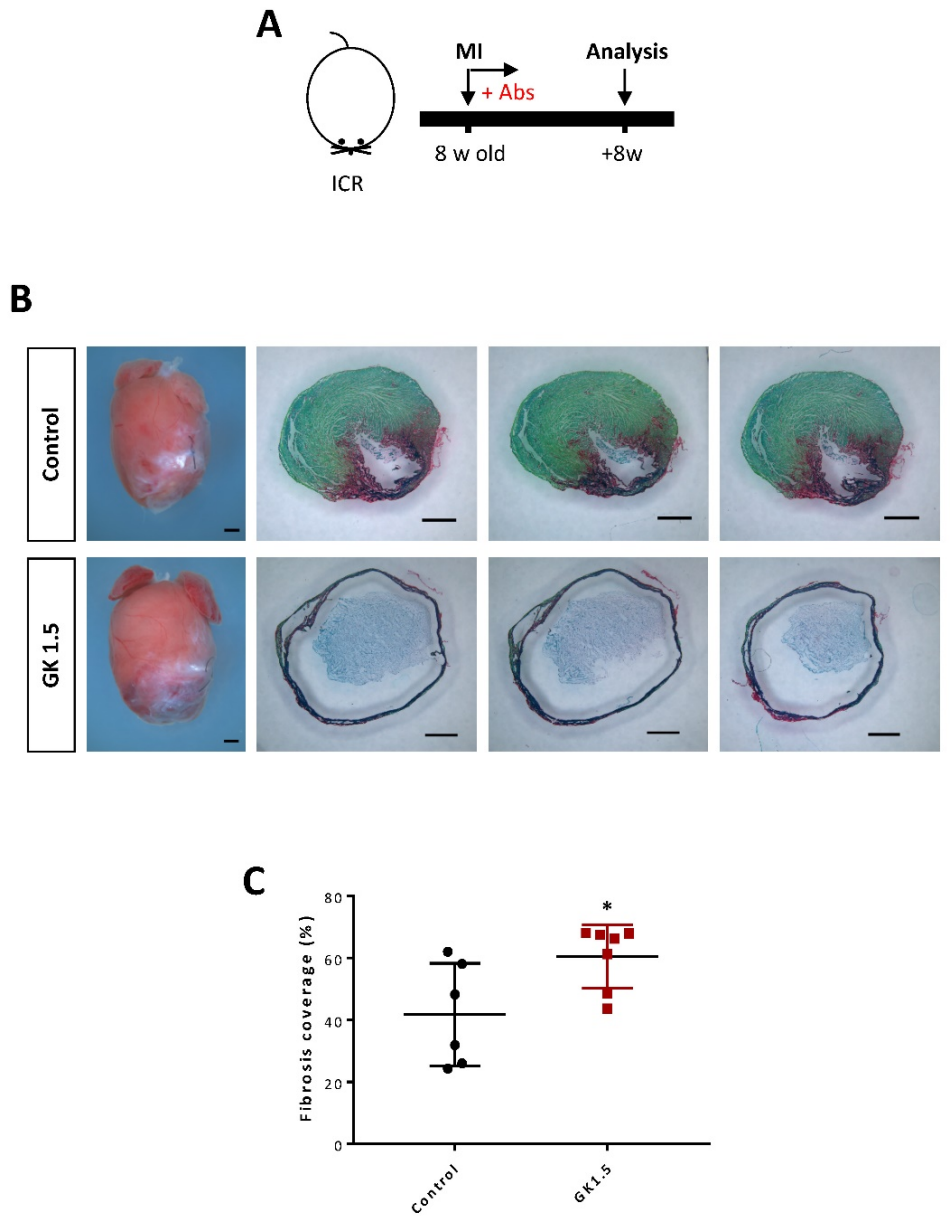
** Ablation of CD4^+^ T-cells did not reactivate adult heart repair after myocardial infarction.** (A) Schematic diagram showing the experimental design. (B) Images of scar tissues, scale bars: 1000 μm; and Sirius red/fast green staining showing representative serial cross sections of fibrotic tissues, scale bars: 1000 μm. (C) Quantification of fibrotic tissue coverage based on (B). Data are presented as mean±S.E.M., *P<0.05, n=6-7 per group.

Previous studies could not conclude the role of CD4^+^ T-cells in adult heart repair as some groups have shown that they are protective 7, 12, 25 while others have revealed that they are detrimental 11, 13 to cardiac repair after myocardial injuries 7, 11, 13. Using the same model through depletion of CD4^+^ T-cells by high dosage of GK1.5 (Figure 7A), we, therefore, asked whether ablation of CD4^+^ T-cells could recapitulate repair of the adult heart in a similar manner as the juvenile heart. At 2 months after MI to the 8 week old ICR mice, we performed Sirius red/fast green staining to determine collagen fibers formed during cardiac fibrosis after myocardial injury (Figure 7B). In contrast to the juvenile heart, treatment with GK1.5 did not contribute to heart regeneration but significantly increased deposition of fibrotic tissues compared to that of the control group (Figure 7C), indicating a protective role of CD4^+^ T-cells in adult heart repair. Altogether, our results may suggest a developmental distinct role of CD4^+^ T-cells in the regulation of heart regeneration and repair.

## Discussion

It has been well established that myocardial injury activates both innate and adaptive immune responses; and, as such, the elevated levels of inflammatory mediators could further compromise cardiac function (for review, see 25, 26). Indeed, heart failure is manifested by a certain degree of chronic inflammation and anti-inflammatory medication such as pentoxifyline has been demonstrated clinical benefit in patients with ischemic and dilated cardiomyopathies 27-29. Nevertheless, there is a lack of therapeutic regimen targeting the adaptive immune response after myocardial injury. Unlike the adult heart, the postnatal murine heart is capable of regenerating shortly after birth till P7 1. It remains unclear whether the regenerating and non-regenerating postnatal hearts display distinct signatures of adaptive immune cells particularly T-cells after injury. Understanding the heterogeneity of different T-cell subsets in the regenerating versus the non-regenerating postnatal heart could further uncover their specific roles in the regulation of heart regeneration.

Within the first two weeks after injury to the non-regenerating P8 heart, there was a significant increase in the absolute number of CD4^+^FOXP3^-^ conventional T-cells per mg heart tissue with a peak at day 7 after injury. Moreover, we observed that there were significantly more conventional T-cells in the non-regenerating P8 than the regenerating P3 heart at day 7 after injury. To ask if these T-cells regulated heart regeneration, we performed loss-of-function experiments. Depletion of CD4^+^ T-cells via the lytic anti-CD4 antibody after CI to the P8 heart reactivated juvenile heart regeneration manifested by significantly reduced cardiac fibrosis and increased number of proliferating cardiomyocytes when compared to that of the control group. Similarly, ablation of CD4^+^ T-cells also reactivated heart regeneration in the P8 heart in an additional AR injury model. Of note, GK1.5 specifically ablated CD4^+^ T-cells including both conventional and regulatory T-cells. As we and others have demonstrated that Treg promote heart regeneration 6, 9, 30, 31, other CD4^+^ T-cell subsets could impede regeneration by some inhibitory mechanisms that were unleashed by treatment with GK1.5. For instances, we revealed that cytokines of Th1 and Th17 directly inhibited the proliferation and promoted the apoptosis of neonatal cardiomyocytes isolated from P1 pups *in vitro*.

Unlike CD4^+^ T-cells, the role of CD8^+^ T-cells in the regulation of heart regeneration and repair has been less clear. We demonstrated that myocardial injury did not recruit more CD8^+^ T-cells to the regenerating P3 or the non-regeneration P8 heart when compared with their respective sham control groups. Moreover, specific ablation by YTS169 did not alter the outcome of heart regeneration when compared to the control group after CI to the P8 heart, suggesting that neonatal/juvenile CD8^+^ T-cells might not play a detrimental role in inhibiting heart regeneration. Under the treatment of GK1.5, the heart still harbored CD8^+^ T-cells but regeneration was resulted. This finding also indicated that either they did not play any role in the regulation of heart regeneration or they needed help from CD4^+^ T-cells to carry out their function. Furthermore, *in vitro* co-culture experiments also revealed that neonatal CD8^+^ T-cells alone were not cytotoxic to cardiomyocytes, in line with a previous finding that the neonatal CD8^+^ T-cells are different from the adult ones as they are not cytotoxic to allogeneic cells after transplantation 32. In our study, the heart still harbored CD4^+^ conventional T-cells and Treg in the YTS169-treated group, and impaired heart regeneration was resulted. We could interpret that the given number of CD4^+^ Treg of the P8 heart were insufficient to promote juvenile heart regeneration whilst conventional T-cells impeded the regeneration. In contrast, deficiency of functional CD8^+^ T-cells leads to improved survival and better cardiac function after MI in the adult CD8a^tm1mak^ mice 33, suggesting a developmentally distinct role of CD8^+^ T-cells in the regulation of heart regeneration and repair.

We also asked if there is a difference in the distribution of various T-cell subsets of the regenerating P3 and non-regenerating P8 heart. Our results of genome-wide, single-cell transcriptomic profiling revealed the heterogeneity of T-cells in response to myocardial injury. We identified both CD4^+^ and CD8^+^ T-cells in the neonatal hearts. Among CD4^+^ T-cells, we found *Ccr7*^+^*Sell*^+^*Cd27*^+^ memory S1 cells, *Foxp3*^+^*Ikzf2*^+^*Nrp1*^+^*Il2ra*^+^ regulatory S2 cells, *Il17a*^+^*Il17re*^+^*Rorc*^+^*Ccr2*^+^ Th17 S3 cells, *Fabp4*^+^*Sparc*^+^ pro-fibrotic S4 cells, chemokine-expressing S5 cells and mitotic gene-expressing proliferating S6 cells. In the adult heart, the IFN-γ^+^ Th1 cells are the predominant effector CD4^+^ T-cells responsible for cardiac fibrosis and dysfunction after injury 8, 23. In the neonatal/juvenile heart, we found more *Il17a*-expressing Th17 cells in addition to *Ifng*-expressing Th1 cells in the non-regenerating compared to the regenerating heart. In addition, the number of pro-fibrotic S4 cells essential for collagen and extracellular fibril organization was tremendously increased in the non-regenerating heart. The S4 cells could, therefore, be the major contributor of cardiac fibrosis in the neonatal heart after injury. Furthermore, the chemotactic S5 cells could also orchestrate chemotaxis of other immune cells including neutrophils and monocytes/macrophages to the injured myocardium.

After injury to the adult heart, there is an influx of M1-like macrophages that promote inflammation, digest necrotic tissues and remove dead cells 34, 35. This is followed by a second influx of M2-like macrophages that resolve inflammation and promote repair through collagen deposition to alleviate ventricular ruptures 34, 35. Treg have been revealed to orchestrate the polarization of M1- to M2-like macrophages during adult heart repair 9. Nevertheless, our recent work showed that depletion of Treg leads to accumulation of M2-like macrophages during scar tissue formation and adoptive transfer of Treg significantly reduces this population during neonatal heart regeneration 6. Here, we further illustrated that treatment with GK1.5 promoted heart regeneration by reducing the polarization of M1- to M2-like macrophages, indicating that CD4^+^ T-cells governed the accumulation of M2-like macrophages in the non-regenerating heart. We interpreted that the discrepancy in the findings of macrophage polarization in the neonatal/juvenile and adult heart could be attributed to developmental factors. Less fibrotic responses are observed in the neonatal/juvenile heart compared to the adult one; and the pro-fibrotic features of M2-like macrophages could be less beneficial for neonatal/juvenile heart regeneration.

In this study, the beneficial effect of CD4^+^ T-cell ablation in the regeneration of the juvenile heart was not recapitulated in the adult heart. The developmental difference in neonatal/juvenile and adult CD4^+^ T-cells could partly contribute to their different responses in the neonatal, juvenile and adult hearts after injury, respectively. For instance, in contrast to adult naïve T-cells that do not normally proliferate unless being transferred to lymphopenic mice, neonatal naïve T-cells proliferate strongly in response to self-peptide/MHC 36, suggesting that they could be more responsive to autoantigens released after injury during the early life. Moreover, unlike their adult counterparts, neonatal naïve T-cells did not convert to a memory-like phenotype 36. In this study, we also showed that the Th1 (TNF-α and IFN-γ) and Th17 (IL-17A) cytokines reduced neonatal cardiomyocyte survival. In fact, adults exhibit more effective Th1- and Th17-type responses than neonates 37. In a model of experimental autoimmune encephalomyelitis, neonates can mount mixed Th1 and Th17-type autoantigen-specific immune responses but there were fewer IL-17-producing Th17 cells in the neonatal than adult mice 38. Furthermore, murine neonatal CD4^+^ thymocytes and T-cells appear to be more readily to differentiate into Foxp3^+^ Treg 39 that can directly promote neonatal heart regeneration given sufficient quantity 6. T-cell receptor-mediated signaling alone is adequate to induce Treg differentiation in neonates without the need of IL-2 and TGF-β that are both required for adult Treg differentiation 39. Therefore, in addition to the binuclear feature of adult cardiomyocytes that exit the cell cycle irreversibly 40, the more effective Th1 and Th17 responses and less effective Treg responses of the adult mice could partly explain that the adult heart is less amenable for repair compared to the neonates. Given the developmental differences in various immune cell types including CD4^+^ T-cells, CD8^+^ T-cells and macrophages, ablation of CD4^+^ T-cells alone was insufficient to promote adult heart repair.

Taken together, our findings have disclosed a previously unrecognized role for CD4^+^ T-cells as regulators of the postnatal heart regeneration, at least in part, by skewing the polarization of pro-fibrotic macrophages, promoting cardiac fibrosis and inhibiting cardiomyocyte proliferation. We have also demonstrated the concept of using immunotherapy in unleashing the inhibitory mechanisms driven by CD4^+^ T-cells for promoting heart regeneration in young mice. We reason that targeting T-cells for tissue regeneration offers an unappreciated advantage particularly the specificity towards neoantigens released from myocardial injury. Our findings could help resolve some of the long-standing questions in the cardiac regeneration field such as how the postnatal heart loses the regenerative capability through development; and what cells negatively regulate the proliferation of cardiomyocytes during heart regeneration in the early life.

## Materials and Methods

### Mice

The *Foxp3^hCD2^* reporter mouse was described previously 6, 14; and ICR was purchased from the Jackson Laboratory. Conventional CD4^+^FOXP3^-^ and CD8^+^ T-cells were depleted via intraperitoneal (i.p.) injection of the lytic anti-CD4 or -CD8 monoclonal antibodies (clones GK1.5 and YTS169, respectively, gifts from Professor Herman Waldmann, University of Oxford) at 0.5 mg/pup or 1 mg/adult on days 0 and 2 after surgery. All animal procedures were approved by the CUHK Animal Experimentation Ethics Committee; and the Institutional Animal Care and Use Committee (IACUC) at the Institute for Nutritional Sciences, Shanghai Institutes for Biological Sciences, Chinese Academy of Science.

### Postnatal mouse heart cryoinfarction

CI was performed as previously described 6. Briefly, neonatal mice at P3 or P8 were subjected to anesthesia by freezing for ∼3-5 minutes (min), and were then placed on the frozen operation table once breathing was steady. Mouse limbs were fixed in location with forceps and 70% ethanol was applied to disinfect the surgical area. An incision (∼1 cm) was made along the sternum and vertical of the chest muscles under a stereomicroscope. Two or three intercostal incisions were made in the left sternum chest to separate the pericardium and expose the left ventricle. Blunt port copper wire (1 mm thickness) was frozen in liquid nitrogen and then put on the left ventricle in order to induce frostbite; this was maintained for ∼7-8 seconds (s) until the left ventricle appeared white. After injury, bubbles and blood in the chest were squeezed out. The chest was closed and the skin was sewn up with 8-0 sutures. After surgery, neonatal mice were placed under a 37°C heating pad to keep warm. They were then placed back with their mothers as soon as they woke up and the skin color returned to normal. In the sham-operated control, we performed the same experimental procedures as above except that we replaced liquid nitrogen with PBS at room temperature.

### Postnatal mouse heart apical resection (AR)

AR was performed as previously described 6. Briefly, neonatal mice at P8 were subjected to anesthesia by freezing for 3-5 min, and were then placed on the frozen operation table once breathing was steady. Mouse limbs were fixed in location with forceps and 70% ethanol was applied to disinfect the surgical area. An incision (∼1 cm) was made along the sternum and vertical of the chest muscles under a stereomicroscope. Two or three intercostal incisions were made in the left sternum chest to separate the pericardium and expose the apex. Curved forceps were extended into the intrathoracic to pull out the heart and the apex was then truncated with microsurgical scissors. After injury, bubbles and blood in the chest were squeezed out. The chest was closed and the skin was sewn up with 8-0 sutures. After surgery, neonatal mice were placed under a 37°C heating pad to keep warm. They were then placed back with their mothers as soon as they woke up and the skin color returned to normal. In the sham-operated control, we performed the same experimental procedures as above except that we did not truncate the heart apex.

### Cell cultures

Murine neonatal cardiomyocytes were isolated with an enzymatic digestion approach as previously described 6. Briefly, neonatal ventricles were minced into small fragments and pre-digested in 0.05% trypsin-EDTA at 4°C overnight. The pre-digested mixture was washed with 10 mM HEPES and 1X penicillin/streptomycin-containing DMEM/F12 medium (light medium) pre-warmed at 37°C, followed by repeated digestions in a stepwise manner: the tissues were digested with 200 U/ml type II collagenase at 37°C for 10 min. After that, the supernatant was collected and mixed in a ratio of 1:1 with DMEM/F12 medium supplemented with 10mM HEPES, 1X penicillin/streptomycin, 10% horse serum (Invitrogen) and 5% fetal bovine serum (dark medium). The supernatant mixture was then kept on ice and the tissue pellet was further digested for 2-3 times with the same procedures until the tissues became single cells. All supernatant mixtures were then pooled together and centrifuged at 800 rpm for 5 min. Differential plating was performed to remove fibroblasts by resuspending the cell pellet with 10 ml dark medium followed by seeding onto a T25 flask at 37°C for 2 hours (h). The unattached cardiomyocytes were then centrifuged at 400 rpm for 5 min, and resuspended with appropriate volume of dark medium for cell counting. Cardiomyocytes were plated on Matrigel (1:100 in DMEM/F12)-coated chamber slide at a density of 10,000 cells per well and cultured in dark medium at 37°C for 24 h. To synchronize proliferation of cardiomyocytes before experiment, they were starved with serum-free medium overnight. After that, they were co-cultured with neonatal CD8^+^ T-cells at a ratio of 1:100 for 3 days. Before co-cultures, neonatal CD45^+^CD3^+^CD8^+^ T-cells were purified from the spleen and activated by 1 ug/ml anti-CD3 (Biolegend), 1 ug/ml anti-CD28 (Biolegend) and 50 ng/ml IL-2 (Peprotech) for 3 days as previously 15. In cytokine treatment experiments, neonatal cardiomyocytes were cultured with 50 ng/ml TNF-α (Peprotech, 300-01A), 50 ng/ml IFN-γ (Peprotech, 300-02) or 100 ng/ml IL-17A (Peprotech, 210-17) at 37°C for 1 day before analysis. For total cardiomyocyte count, cardiomyocytes were cultured with IFN-γ, TNF-α or IL-17A for 3 days and harvested at a single-cell level by enzymatic digestion with 0.25% trypsin-EDTA for at 37°C for 5 min before centrifugation.

### Immunostaining

Cells cultured *in vitro* were fixed in 4% paraformaldehyde at room temperature for 25 min or heart tissues were dissected and fixed in 4% paraformaldehyde at 4°C overnight. The fixed heart tissues were washed three times with PBS and equilibrated in 30% sucrose at 4°C for 2 days before freezing and cryosectioning. Eight micrometer frozen sections were prepared. Cells or tissue sections were blocked with 5% BSA and 5% goat serum and then stained with the respective primary antibodies at 10 ug/ml at 4˚C overnight. Anti-mouse primary antibodies used: COLA1 (abcam, ab34710), cTnT (abcam, ab8295), pH3 (Millipore, 06-570), Ki67 (abcam, ab15580) or cCASP3 (Cell signaling technology, 9661). Alexa-Fluor-488- or Alexa-Fluor-546-conjugated secondary antibodies (Invitrogen) were used at room temperature for 30 min in the dark. For cell death analysis, an *in situ* cell death detection kit by TUNEL was also used per manufacturer's instruction (Roche, 1256792910). Slides were mounted with DAPI-containing fluorescence mounting medium (Dako) and fluorescence was detected with an upright fluorescence microscope, inverted fluorescence microscope or confocal microscope (all Leica). Images were processed with the ImageJ software and cTNT coverage was analyzed based on this formula: cTNT^+^ area/total area.

### Masson's trichrome staining and Sirius red/fast green staining for collagen fibers

Masson's trichrome staining was performed to determine collagen deposition per manusfacturer's instruction (Polysciences 25088-1). Briefly, frozen sections were washed in PBS and fixed in 4% paraformaldehyde for 8 min. Slides were then incubated in Bouins' solution (5% acetic acid, 9% formaldehyde and 0.9% picric acid) at room temperature overnight. The next day after washed with distilled water, slides were incubated in Weigert's iron hematoxylin solution for 10 min, washed and then stained with Biebrich scarlet-acid fuchsin for 5 min. After three washes with distilled water, slides were incubated in phosphotungstic/phosphomolybdic acid for 10 min followed by staining with aniline blue solution for 5 min. After that, slides were washed with distilled water for three times and dehydrated with ethanol and xylene based on standard procedures. For Sirius red/fast green staining, sections were fixed with prewarmed Bouin's solution at 55°C for 1 h and then rinsed in running water. Sections were stained in 0.1% fast green solution for 10 min and then washed with 1% acetic acid for 2 min. After rinsing in tape water, sections were stained in 0.1% Sirius solution for 30 min. After staining, sections were dehydrated and cleared with xylene. Images were acquired on Nikon eclipse TE2000-S microscope. Images were analyzed by the ImageJ software. For the CI group, fibrosis coverage was quantified based on this formula: scar perimeter/total perimeter; and for the AR group, scarring was quantified based on this formula: area of the fibrotic scar/total area of the ventricle.

### Flow cytometry, cell sorting and analysis

To study immune cell infiltrates in the postnatal heart, heart tissues were minced into small fragments and dissociated with 1:1 type II collagenase (1000 U/ml in PBS, Worthington) and dispase (11 U/ml in PBS, Gibco) at 37°C for 30 min. Enzymatic action was stopped by adding 10% FBS and the dissociated cells were washed twice with PBS. The dissociated single splenocytes or neonatal heart cells were removed from the contaminated erythrocytes by incubating with the red blood cell lysis buffer (eBiosciences) for 5 min; and were then blocked with 2% normal rabbit serum. Cells were subsequently stained with fluorochrome-conjugated antibodies against the following antigens: CD3, CD4, CD8, CD45, CD206, F4/80 or Ly6C (Biolegend or eBiosciences) at a dilution of 1:100, unless specified by the manufacturer, at 4°C for 30 min. Murine Treg of ICR mice were detected with the Treg staining kit according to manusfacturer's instructions (eBioscience). Cells were then washed three times with 2% FBS-containing PBS and analyzed on flow cytometer (BD FACSAria^TM^ Fusion). Propidium iodide (PI, BD) positive dead cells were excluded for live cell analysis/sorting; and FACS data were then analyzed with the FlowJo software (Tree star).

### Single-cell encapsulation and library preparation

Single cells were purified by FACS sorting before library preparation and single-cell libraries were prepared with the Chromium Single Cell 3' Reagent Kits v2 (10x Genomics) as per manufacturer's instructions. Briefly, sorted cells in suspension were first prepared as gel beads in emulsion (GEMs) on Single Cell 3' Chips v2 (10x Chromium) using the Chromium Controller (10x Genomics). Barcoded RNA transcripts in each single cell were reverse transcribed within GEM droplets. cDNA was purified with DynaBeads MyOne Silane beads (Invitrogen) and then amplified for subsequent library construction. Sequencing libraries were prepared by fragmentation, end-repair, ligation with indexed adapters and PCR amplification using the Chromium Single Cell 3' library kit v2 (10x Genomics). Nucleic acid was cleaned up after each steps using SPRIselect beads (Beckman Coulter). Libraries were then quantified by Qubit and real-time quantitative PCR on a LightCycler 96 System (Roche).

### Single-cell RNA-sequencing and functional annotations

Pooled libraries were sequenced on the Illumina HiSeq 2500 platform. All single-cell libraries were sequenced with a customized paired-end dual index format (98/26/0/8 basepair) according to manufacturer's instructions. Data were processed, aligned and quantified using the Cell Ranger Single-Cell Software Suite (v 2.1.1). Briefly, data were demultiplexed based on the 8 base-pair sample index, 16 base-pair Chromium barcodes and 10 base-pair unique molecular identifiers (UMI). After quality control, reads were aligned on *Mus Musculus* Cell Ranger transcriptome reference (mm10-1.2.0). To eliminate the impact of cell number bias, data from 1,000 cells of each sample were randomly selected for further analysis as previously demonstrated 14. Data analyses, including tSNE and graph-based clustering, were performed according to Cell Ranger's pipelines with default settings. Cells were filtered for subsequent analysis if their mRNA contents were above or below two standard deviations of the mean value; if they showed high mitochondrial contents as demonstrated by fractions of mitochondrial transcripts > 10%; or if they expressed B cell specific markers *Ebf1*, *Cd79a* and *Cd79b*. Differentially expressed genes in each cluster relative to all other clusters were identified by Cell Ranger's pipelines with default settings (minimum mean expression = 0.01 and adjusted p-value cutoff = 0.05). Gene ontology (GO) enrichment analyses of the cluster-specific highly expressed genes were performed by DAVID Bioinformatics Resources (v6.8).

### Real-time quantitative PCR (RT-qPCR)

Total RNA was isolated from flow cytometer-sorted live cells using RNeasy mini kit (Qiagen) and reverse transcribed using iScript cDNA synthesis kit (Bio-Rad), according to manufacturers' instructions. RT-qPCR was analysed on QuantStudio^TM^ 12K Flex Read-Time PCR system (ThermoFisher Scientific) via SYBR Green (Bio-Rad). Values for specific genes were normalized to the housekeeping controls, *beta-actin* and *Gapdh*. The relative gene expression level of each sample was also compared with an internal control.

### Statistical analysis

The data were expressed as arithmetic mean±S.E.M. of biological replicates (n = 5, unless otherwise specified) performed under the same conditions. Statistical analysis was performed using the unpaired student's t-test with data from two groups; while date from more than two groups was performed using an ANOVA followed by Tukey's method for multiple comparisons. Significance was accepted when *P* < 0.05.

## Supplementary Material

Supplementary figures and tables.

## Figures and Tables

**Table 1:** ** Distribution of cell number and percentage of CD3^+^ T-cells in each of the six subsets as determined by t-SNE.** At day 7 after CI, we purified CD45^+^CD3^+^ T-cells from the hearts of ICR mice that underwent CI at P3 and P8 by flow cytometry and captured the transcriptome of about ~1,123 and ~2,167 cells by scRNA-seq, respectively. Cells were filtered out if their mRNA contents were above or below two standard deviations of the mean value; if they showed high mitochondrial contents as demonstrated by fractions of mitochondrial transcripts > 10%; or if they expressed B cell specific markers *Ebf1*, *Cd79a* and *Cd79b*. After that, ~1,000 cells were randomly selected from each sample for further analysis.

	Total	P3	P8
**After filtered**	3290	1123	2167
**Randomly selected**	2000	1000	1000
**S1**	1437	764 (76.4%)	673 (67.3%)
**S2**	190	100 (10.0%)	90 (9.0%)
**S3**	157	65 (6.5%)	92 (9.2%)
**S4**	105	3 (0.3%)	102 (10.2%)
**S5**	78	43 (4.3%)	35 (3.5%)
**S6**	33	25 (2.5%)	8 (0.8%)
