# Supplementary material for: Specific ablation of CD4+ T-cells promotes heart regeneration in juvenile mice
Source: Theranostics. 2020 Jun 29;10(18):8018–35. doi: 10.7150/thno.42943 (PMC7381734; doi:10.7150/thno.42943)
Supplement: Supplementary file 1 — Supplementary figures and tables. [file thnov10p8018s1.pdf]

Supplementary Figures

Figure S1 Depleting efficiency of GK1.5 and YTS169 targeting $CD4^+$ and $CD8^+$ T-cells, respectively, after neonatal heart injury. Flow cytometric analysis and quantifications showing %$CD3^+CD4^+$ and %$CD3^+CD8^+$ T-cells among total peripheral blood cells at (A) 7 days or (B) 4 weeks after CI of ICR mice treated with anti-CD4 (GK1.5) and anti-CD8 (YTS169) monoclonal antibodies, respectively. Data are presented as mean±S.E.M., ***$P<0.001$, $n=6$ per group.

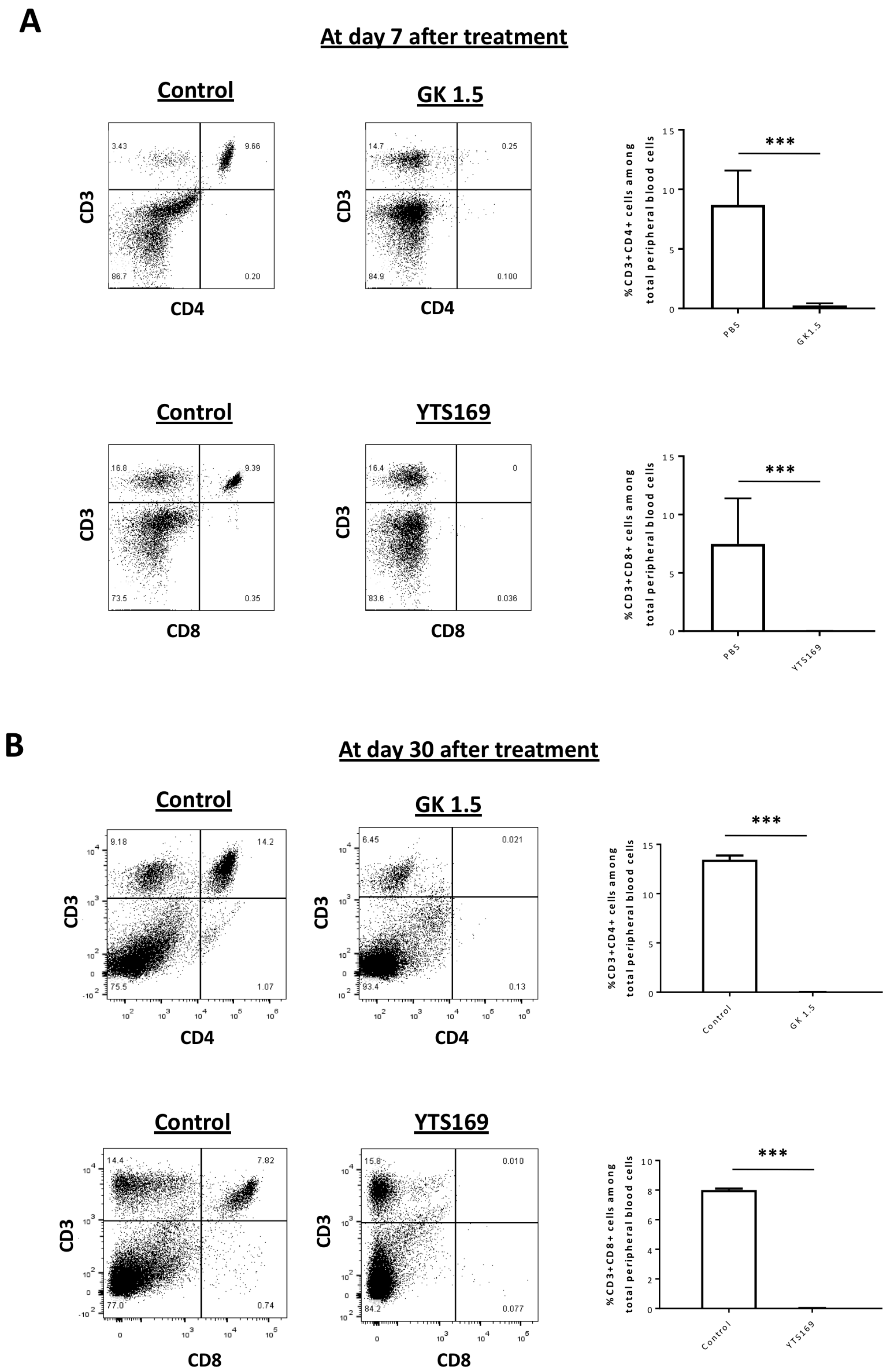
A
At day 7 after treatment
Control
GK 1.5
CD3
CD4
3.43
9.66
86.7
0.20
14.7
0.25
84.9
0.100
% CD3+CD4+ cells among total peripheral blood cells
***
PBS
GK1.5
Control
YTS169
CD3
CD8
16.8
9.39
73.5
0.35
16.4
0
83.6
0.036
% CD3+CD8+ cells among total peripheral blood cells
***
PBS
YTS169
B
At day 30 after treatment
Control
GK 1.5
CD3
CD4
9.18
14.2
75.5
1.07
6.45
0.021
93.4
0.13
% CD3+CD4+ cells among total peripheral blood cells
***
Control
GK 1.5
Control
YTS169
CD3
CD8
14.4
7.82
77.0
0.74
15.8
0.010
84.2
0.077
% CD3+CD8+ cells among total peripheral blood cells
***
Control
YTS169

Figure S2 Neonatal CD8$^+$ T-cells are not cytotoxic to cardiomyocytes. Neonatal CD8$^+$ T-cells were co-cultured with neonatal cardiomyocytes at a ratio of 1:100 for 3 days. (A). Immunostaining for pH3$^+$ (green) and cTnT$^+$ (red) cells; or cCASP3$^+$ (green) and cTnT$^+$ (red) cells, scale bars: 50 μm. Arrows indicate cardiomyocytes positive for pH3 or cCASP3. (B, C) Quantification of absolute number of (B) %pH3$^+$cTnT$^+$ or (C) %cCASP3$^+$cTnT$^+$ cardiomyocytes among total cTnT$^+$ cardiomyocytes. Data are presented as mean±S.E.M., n = 3 independent experiments.

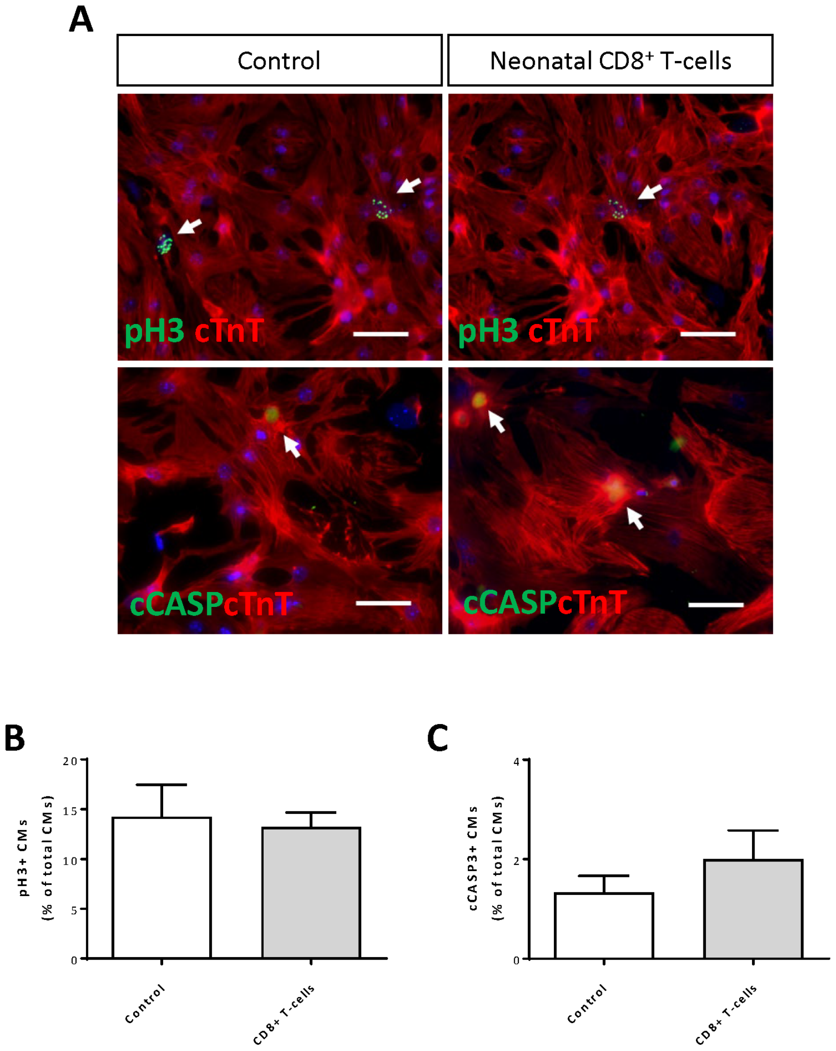

Figure S3 *t*-SNE plots confirm the purity of purified T-cells of the neonatal hearts for single-cell transcriptomic profiling. CD45^{+}CD3^{+} T-cells were purified from the hearts of ICR mice that underwent CI at P3 and P8, respectively, by flow cytometry. Most cells express the *Cd3* transcripts with signature of CD4^{+} (*Cd4*, *Zbtb7b*) or CD8^{+} (*Cd8a*, *Cd8b1*) T-cells.

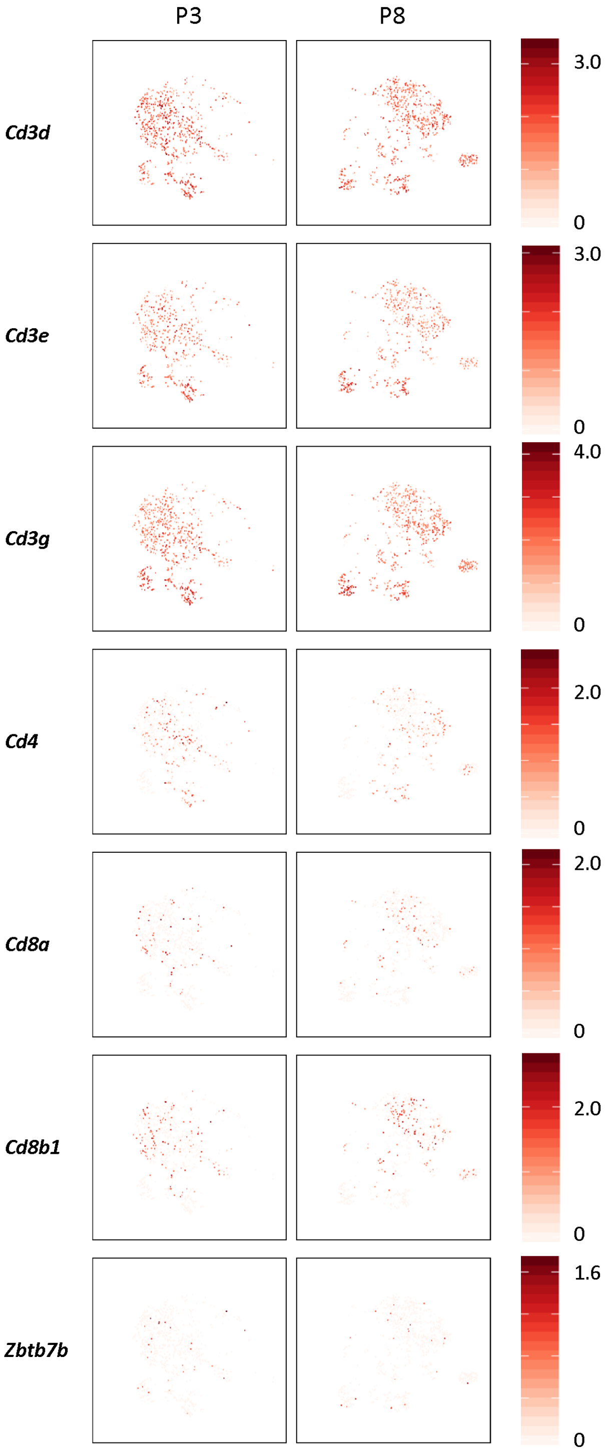

Figure S4 *t*-SNE plots showing expression of cytotoxic genes by CD3$^+$ T-cells of the neonatal hearts after injury. CD45$^+$CD3$^+$ T-cells were purified from the hearts of ICR mice that underwent CI at P3 and P8, respectively, by flow cytometry. *Gzmb* was the predominantly expressed cytotoxic gene in cells of S3. Although few cells displayed cytotoxicity, more cells of the P8 than P3 hearts expressed these cytotoxic genes.

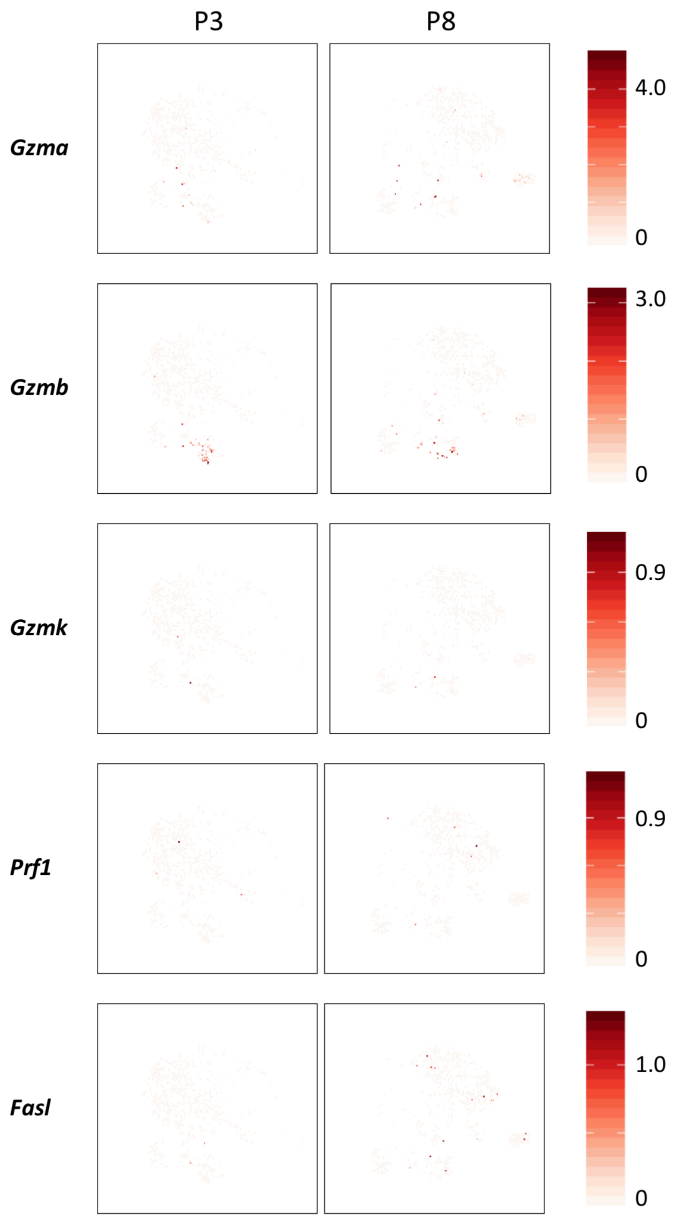

Figure S5 *t*-SNE plots showing expression of memory cell associated genes by $CD3^+$ T-cells of the neonatal hearts after injury. $CD45^+CD3^+$ T-cells were purified from the hearts of ICR mice that underwent CI at P3 and P8, respectively, by flow cytometry.

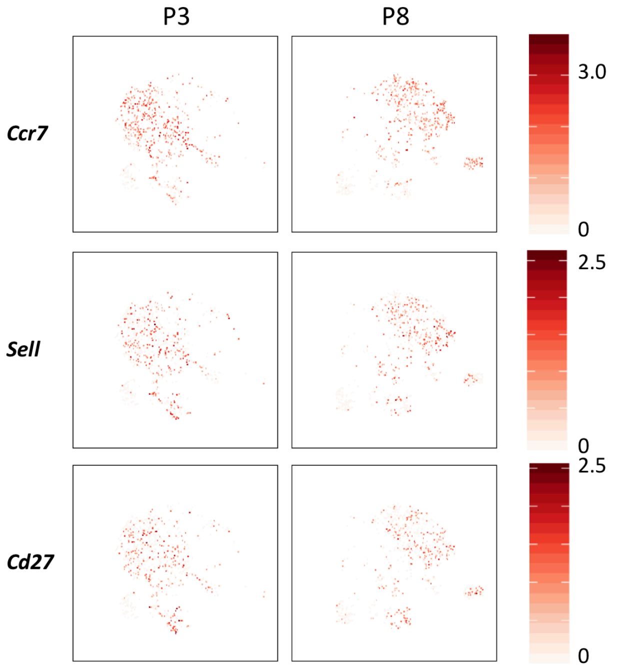

Figure S6 *t*-SNE plots showing expression of genes specific for various T-cell subsets of the neonatal hearts after injury. CD45$^+$CD3$^+$ T-cells were purified from the hearts of ICR mice that underwent CI at P3 and P8, respectively, by flow cytometry. (A) Genes are specific for Treg; (B) for Th17; (C) for Th1 and (D) for Th2 cells.

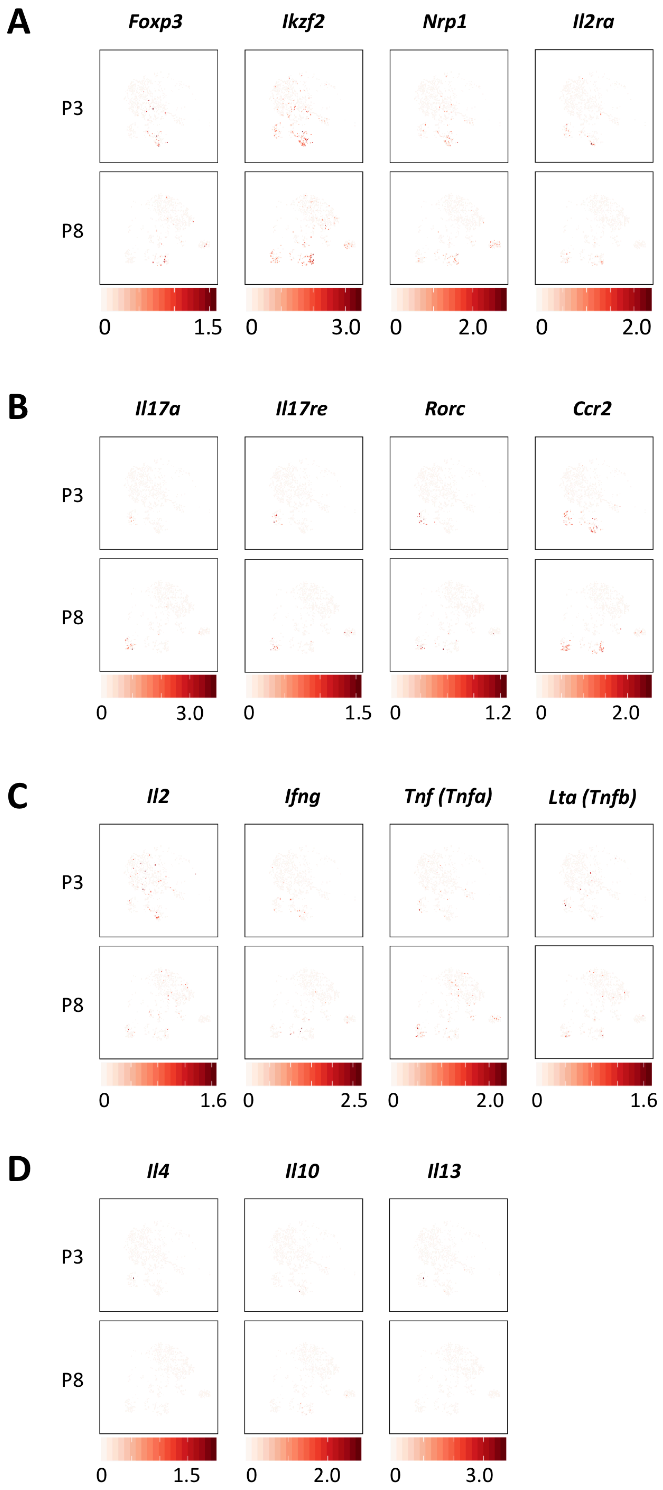

Fig S7 Cytokines of Th1 and Th17 cells are cytotoxic to neonatal cardiomyocytes. (A) Immunocytochemistry for cTnT$^+$ (green) and TUNEL$^+$ (red) cells showing cell death of neonatal cardiomyocytes after cultured with PBS (solvent control), 50 ng/ml TNF-α, 50 ng/ml IFN-γ or 100 ng/ml IL-17A for 1 day, scale bars: 50 μm. White arrows indicate apoptotic TUNEL$^+$cTnT$^+$ cells; and squares showing enlarged nuclei positive for TUNEL. (B) Quantification of (A) showing %TUNEL$^+$cTnT$^+$ cardiomyocytes among total cTnT$^+$ cardiomyocytes. Data are presented as mean±S.E.M., n = 3 independent experiments with a total of ~20,000 cardiomyocytes counted, *P<0.05, **P<0.01.

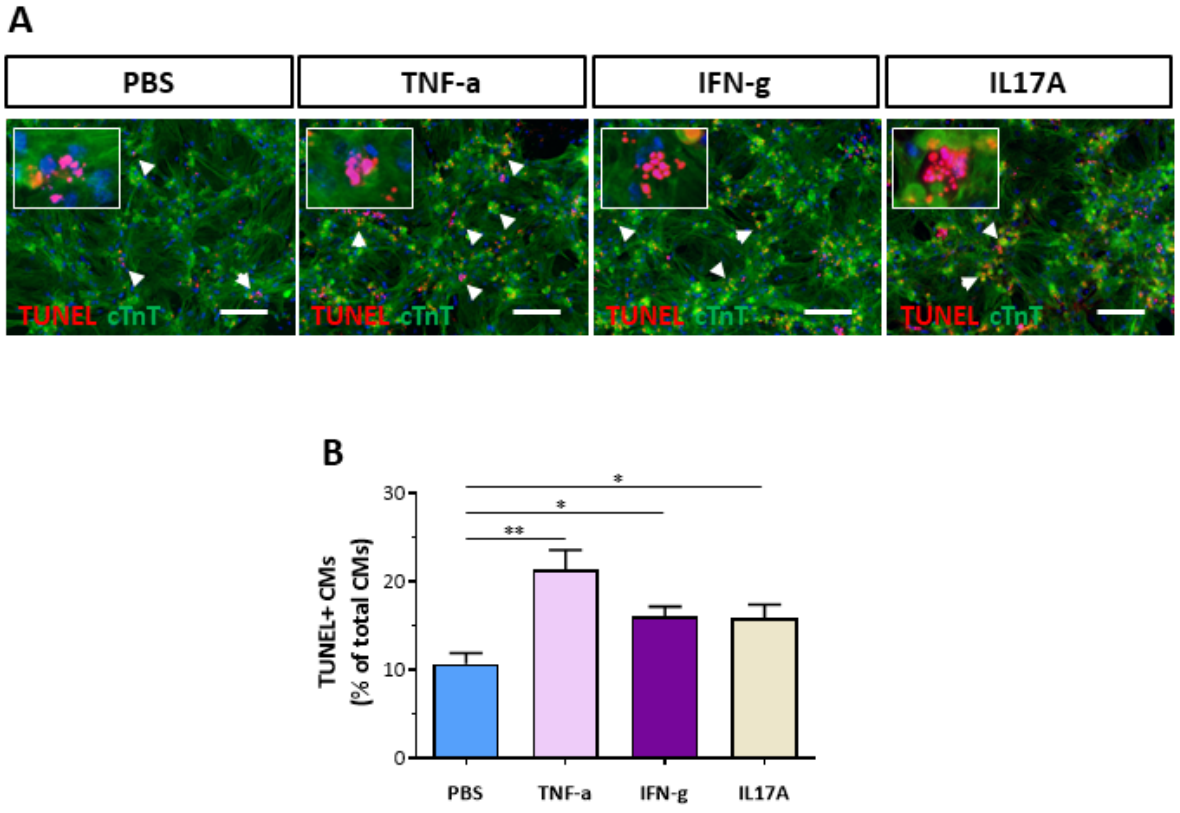

Supplementary Tables

Table S1 A list of subset-specific genes as identified by *t*-SNE analysis.

Clusters	Genes
**S1**	Dapl1, Satb1, Tdrp, Cd8b1, Igfbp4, Lef1, Pik3ip1, Ntrk3, Slfn1, Tcf7, Bach2, Tubb2a, Ccr7, Cd55, Bcl2, Foxp1, Cd8a, Tmem108, Gm8369, Rab3ip, Dusp10, Atp1b1, Klhdc2, Themis, Ssbp2, Itm2a, Actn1, Sidt1, Ubxn11, Ibtk, Pptc7, RP23-6I17.1, Treml2, Ttc3, Dgka, Enc1, Txk, Klhdc1, Il6ra, Smchd1, Fam101b, Map7, Cdkn2aip, Gm12840, Ssh2, Rasgrp2, 4932422M17Rik, Dtx1
**S2**	Klrg1, Gzmb, Ctla4, AW112010, Il1rl1, Tnfrsf4, Ikzf2, Itgb1, Icos, Sdcbp2, Samsn1, S100a6, Hopx, Gna15, S100a11, S100a4, Glrx, Pglyrp1, Foxp3, Tnfrsf18, Rgs1, Nrp1, Rgs16, Ass1, S100a10, Tnfrsf9, Sh3bgrl, Rora, Crip1, Capg, Egln3, Lgals1, Vim, Cmtm7, Acot7, Lgals3, Prr13, Atxn1, Sla, Ifi27l2a, Stx11, Cd48, Cxcr3, Gbp3, Slc25a19, Il2ra, Cd83, Ccr2, Actg1, Ppil1, Socs2, Traf1, Ebp, Hilpda, Osbpl3, Pmaip1, Mif4gd, Matk, Gm2a, Gimap7, Slc4a7, Izumo1r, Pkp3, Dusp4, Ifng, Cd200r1, Slc16a3, 2010111I01Rik, Tnfrsf1b, Tstd3, Dnajc15, Phf11b, Impa2, Lamtor5, Il2rb, Cd274, Atp2b4, Serinc3, Itgav, Myo1f, Pear1, Agpat4, Epsti1, Fgl2, Psen2, Plekhb2, Fam129a, Gbp7, Acadl, Tmem173, Coro2a, Mthfsl, Map2k3, Cldnd1, Rln3, Ahnak, Lmna, Ubash3b, Maf, Adora2a, Pqlc3, 1500009L16Rik, Batf, Anxa2, Raph1, Peak1, Eea1, Bcl2a1b, Reep5, Vamp8, Tank, Sytl1, Pycard, Trp53i11, Srgn, Etfb, Rab8b, Lsp1, Nfil3, Rom1, Fuca2, Vmp1, Crem, Slc22a15, Gem, Alad, Aim2, Mmd, Itgb7, Rab19, Myadm, Tgif1, BC021614, Ebpl, Rhoc, Snrnp25, Oaz2, Arl13b, Icam1, Tkt, Glmp, Smpdl3a, Mdh1, Sar1b, Vps54, Arl5a, Plp2, Tnfaip8, Capn2, Snx2, Cd5, Ndrg1, Glipr1, Lcp1, Plin2, Fkbp1a, S100a13, Ncf4, AU020206, Gpr68, Blmh, Dhrs7, Smap1, Bcl2a1d, Ly6e, Ifrd1, Mir155hg, Ptprcap, Snx18, Smco4, Hcls1, Mien1, Sytl2, Tagln2, Dnaaf5, Rilpl2, Pla2g16, Abracl, Dok2, Ccl5, Isg20, Tpi1, Atp2b1, Ctsa, Pef1, Cyb5a, Cdc42, Ift52, Ccdc69, Nfkb1, Cbr4, Csrp2, Cpm, Cd6, Nkg7, Sdhaf2, Ybx3, Cap1, Sh2d1a, Unc119, Smim24, Ndufb7, Dgcr6, Mrpl40, Lpxn, H2afz, Itm2b, Ubl3, Faim, Ccrl2, Etaa1os, Cox17, Actb, Dok1, Zc3h12d, Ube2l3, Mpc1, Cmpk1, Ikzf3, Syngr2, Prdx4, Pim1, Acsbg1, Ptpn7, Tspo, Ppp1ca, Pigs, Dpp3, H2-D1, Hsd11b1, Arid3a, Mrpl54, Bhlhe40, Cd2, Arpc5, Gyg, Prelid1, Trpv2, Crlf2, Rpap3, Lamtor4, Crip2, Ptger4
**S3**	Cd163l1, Cxcr6, Tmem176a, Ly6g5b, Blk, Kcnk1, Serpinb1a, Tmem176b, Ltb4r1, 5430421N21Rik, Il1r1, Abi3bp, Tns4, Ccr2, S100a4, Il17re, Slc6a13, S100a6, Podnl1, Hbegf, Rorc, F2r, Il18r1, Bcl2a1d, Aqp3, Zbtb16, St6galnac3, Avpi1, Il17a, Tnfsf14, Maf, Cd82, Bcl2a1b, Lgals3, Fgl2, Bhlhe40, Hk2, Cd44, Ramp1, Capg, Sept11, Klrk1, Pdcd1, Il12rb1, St3gal6, S100a11, Atp2b4, Rora, Rgs1, Sox13, Adgrg5, Bcl2a1a, Acsbg1, Slc16a6, Gm1673, 1500009L16Rik, Myo1e, Tnf, Tasp1, Ckb, Amica1, Acot7, Lxn, Ramp3, Lmo4, Ly6e, Scpep1, Gpr183, Fam129a, Id2, Naga, Znrd1, Mgat4a, Lgals1, Fam110a, Furin, Mdfic, Tppp3, Comt, Ahnak, Gabbr1, Cish, Mycn, Socs2, Sptssa, Cdkn1a, Rnase4, Osbpl3, Nebl, Sdf2l1, Gm29243, Tnfaip8, Antxr2, Sdccag8, Zfp36l1, Sec11c,

	Hnrnpll, Cst7, Plekho2, Lax1, Hilpda, Prr13, Mgat5, Znrf1, Lta, Irf5, Prelid2, Fam213a, Gng2, Plxnd1, Sdc4, Ikzf3, Xlr4c, Eif4ebp1, Il2ra, Rnh1, Cd226, Adam8, Cd247, Unc119, Anxa1, Smox, Emp1, Abi3, Zap70, Wbp1, Anxa2, Wls, Eml2, Nav2, Mfsd10, Fnbp1, Abhd16a, Il18rap, Gramd1b, Dnaja4, Smpdl3a, Zyx, Serpinb6a, Cxcr3, Cit, Cd164, Xlr4a, Nedd4, Fosb, Cers4, Nucb1, Dap, Gpatch2, Rarg, Pdia6, Cmtm7, Ppp1r11, Rrad, Igf1r, Lgals3bp, S100a10, Ncf4, Diaph1, Tagln2, Fkbp2, Rinl, D16Ertd472e, Laptm4b, Pi4k2a, Psap, Eif4e3, Nabp1, Gch1, Ccdc50, Ppp3ca, Clcn3
**S4**	S100a8, Fabp4, Sparc, Mgp, S100a9, Dcn, Lum, Eln, Gsn, Mfap4, Mfap5, BC100530, Rbp7, Gng11, Col3a1, Col1a1, Egfl7, Tm4sf1, Col1a2, Cd36, Lpl, Aspn, Gm5483, Bgn, Gpihbp1, Cxcl12, Ly6c1, Cav1, Col4a2, Sparcl1, Ltbp4, Crip2, Rarres2, Cd34, Sdpr, Lyl1, Dpt, Pam, Mgll, Myl9, Fstl1, Pmp22, Nenf, Myct1, Fabp5, Ifitm3, Pcolce, Hspg2, Ppic, Selm, S100a16, Gpx7, Tcf4, Cald1, Rgs5, Ifitm2, Gpx3, Ccl9, Serpinh1, Anxa3, Col4a1, Lhfp, Pid1, Csrp2, Cnn3, Cd63, Cd81, H2-Ab1, Ccl7, Plk2, Erdr1, Hspb1, Cdh5, Spry2, Esam, Pkig, Mmp2, Id1, Rhoc, Ptn, Pi16, Fn1, Cst3, Cyr61, Dusp3, Ier3, Bmpr2, Hmox1
**S5**	Lyz2, Apoe, Pf4, C1qa, Fcer1g, Tyrobp, C1qb, C1qc, Hpgd, Dab2, Fcgr3, Mef2c, Cxcl16, Cx3cr1, Gatm, Csf1r, Pla2g7, Aif1, F13a1, Spi1, Cd63, Wfdc17, Mt1, Zeb2, Cst3, Cd14, Alox5ap, Trf, Ctsh, Lst1, Lyn, Cd68, Clec4n, Ccl2, Mrc1, Fcrls, C5ar1, Cbr2, Ifitm2, Unc93b1, Pld4, Cxcl2, Clec4a2, Lgmn, Blvrb, Ccl6, Cybb, Ly86, Slc40a1, Grn, Adgre1, Sirpa, Ifitm3, Ltc4s, Igf1, Ccl12, Camk1, Lilrb4a, Stab1, Ctsb, Hpgds, Hmox1, Ms4a7, Ehd4, Ctsz, Ctsc, Gas7, Lepr, Marcks, Ms4a6d, Hexa, Ms4a6c, Skap2, Asah1, Ctss, Rnpep, Sepp1, Atf3, S100a1, Abhd12, Plek, Cd86, Dhrs3, App, Tpd52, Fcgrt, Marcksl1, Ccrl2, Rnase4, Il1b, Ccl4, Pltp, Efhd2, Hebp1, Ccl3, Sh2b3, Ccnd1, Pid1, Cd83, Ier3, Ccl9, Ccl7, Lamp2, Pmp22, Dusp3, Ralb, Lamp1, Cxcl10, Ifi30, Ckb, Cxcl1, Soat1, Apobec1, Plin2, H2-DMa, Pkig, Hexb, Ncf1, Atp6v1a, Tm6sf1, Nfic, Cd74, Irf5, Tctex1d2, Ftl1, Slc25a10, Plac8, Cyr61, Gstm1, Anxa5, Irf8, Cd81
**S6**	2810417H13Rik, Top2a, Stmn1, Asf1b, Birc5, Rrm2, Ube2c, Cks1b, Cdca3, Mki67, Tuba1b, Ccnb2, Smc2, Incenp, Tyms, Fbxo5, Tacc3, Cenpm, Cdca8, Spc24, Nusap1, Cenpf, Aurka, Prc1, Mad2l1, Ccdc34, Hmgb2, Hmmr, Cenpe, Lockd, Mcm5, Pttg1, Tubb5, Knstrn, Pmf1, Hmgn2, Cdc20, Lig1, Racgap1, Cenpw, Lmnb1, Nrm, H2afx, Orc6, Mcm3, Arhgap11a, Tipin, Cenpa, Prim1, Dut, Ncapd2, H2afv, Hells, G2e3, Syce2, Kif20b, Cdc25b, Cdca7, Dhfr, Cks2, Hmgb1, Tuba1c, 2700094K13Rik, Cmc2, H2afz, Dlgap5, Mcm6, Kif22, Rrm1, Rfc2, 4930579G24Rik, Ranbp1, H1f0, Ezh2, Lsm2, Gmnn, Cit, Hist1h2bc, Banf1, Haus1, Tmpo, Prim2, Nap1l1, Tmem43, Txn1, Ska2, Anxa2, Nutf2-ps1, Ptma, Usp1

Table S2 GO enrichment analysis showing the selected, most significant biological processes in cells of each subset of the $CD3^+$ T-cells purified from the regenerating and non-regenerating neonatal heart, respectively, after injury.

Clusters	P value	GO Term	Genes
**S1**	0.002434485	GO:0042110~T cell activation	Satb1, Cd8a, Treml2
	0.003251498	GO:0006470~protein dephosphorylation	Bcl2, Ssh2, Dusp10, Pptc7
	0.003318299	GO:0002250~adaptive immune response	Cd8b1, Cd8a, Themis, Txk
**S2**	1.22E-05	GO:0006915~apoptotic process	Traf1, Trpv2, Lgals1, Pim1, Pglyrp1, Egln3, Nfkb1, Gzmb, Pmaip1, Mien1, Aim2, Lsp1, Prelid1, Tnfrsf9, Tmem173, Tnfaip8, Ifng, Pycard, Tnfrsf18, Faim, Srgn
	7.07E-05	GO:0050728~negative regulation of inflammatory response	Tnfrsf1b, Il2ra, Ptger4, Adora2a, Pglyrp1, Nfkb1, Rora, Foxp3
	6.43E-04	GO:0050710~negative regulation of cytokine secretion	Ptger4, Foxp3, Tnfrsf4, Srgn
**S3**	5.91E-06	GO:0032729~positive regulation of interferon-gamma production	Il18r1, Il12rb1, Tnf, Ccr2, Klrk1, Cd226, Lta
	5.09E-05	GO:0006954~inflammatory response	Tnf, Lxn, Anxa1, Il17re, Cxcr3, Il17a, Ltb4r1, Cxcr6, Ccr2, Zap70, Adam8, Lta, F2r
	2.99E-04	GO:0019221~cytokine-mediated signaling pathway	Il1r1, Podnl1, Irf5, Socs2, Cd44, Ccr2, Il17re, Cish
**S4**	8.13E-07	GO:0030199~collagen fibril organization	Lum, Col3a1, Col1a2, Col1a1, Serpinh1, Dpt
	3.98E-06	GO:0042060~wound healing	Cav1, S100a8, Col3a1, Col1a1, Sparc, Dcn, Fn1
	1.39E-05	GO:0043206~extracellular fibril organization	Col3a1, Cst3, Mfap4, Mfap5
**S5**	2.60E-15	GO:0006935~chemotaxis	Ccl3, Ccl2, C5ar1, Lyn, Cxcl2, Ccl9, Pf4, Ccl4, Ccl7, Ccl6, Cxcl10, Ccrl2, Ccl12, Cxcl16, Cx3cr1, Cyr61
	3.61E-14	GO:0030593~neutrophil chemotaxis	Cxcl1, Ccl3, Ccl2, C5ar1, Cxcl2, Ccl9, Ccl4, Ccl7, Ccl6, Fcgr3, Ccl12, Fcer1g, Il1b

	3.31E-12	GO:0070098~chemokine-mediated signaling pathway	Cxcl1, Ccl12, Ccl3, Ccl2, Cxcl2, Ccl9, Pf4, Ccl4, Ccl7, Ccl6, Cxcl10
**S6**	1.20E-34	GO:0007049~cell cycle	Cks1b, Prc1, Haus1, Tipin, Aurka, Pttg1, Spc24, Cdca8, Incenp, Fbxo5, Ska2, H2afx, Hells, Cdca3, Mki67, Gmnn, Lig1, Dlgap5, Nusap1, Syce2, Cenpe, Cdc20, Birc5, Pmf1, Racgap1, Knstrn, Mcm3, Ube2c, Smc2, Mcm5, Mcm6, Cdc25b, Ncapd2, Ccnb2, Mad2l1, Kif20b, Cks2, Cenpw, Cit
	2.38E-26	GO:0007067~mitotic nuclear division	Cks1b, Prc1, Haus1, Tipin, Aurka, Pttg1, Spc24, Cdca8, Incenp, Fbxo5, Ska2, H2afx, Hells, Cdca3, Mki67, Gmnn, Lig1, Dlgap5, Nusap1, Syce2, Cenpe, Cdc20, Birc5, Pmf1, Racgap1, Knstrn, Mcm3, Ube2c, Smc2, Mcm5, Mcm6, Cdc25b, Ncapd2, Ccnb2, Mad2l1, Kif20b, Cks2, Cenpw, Cit
	1.61E-10	GO:0006260~DNA replication	Prim1, Rfc2, Lig1, Rrm2, Prim2, Rrm1, Orc6, Mcm3, 2810417h13rik, Mcm5, Mcm6
